# Supplementary figures and images for: Monitoring bay-scale ecosystem changes in bivalve aquaculture embayments using flow cytometry
Source: PLoS One. 2024 Nov 5;19(11):e0313271. doi: 10.1371/journal.pone.0313271 (PMC11537403; doi:10.1371/journal.pone.0313271)

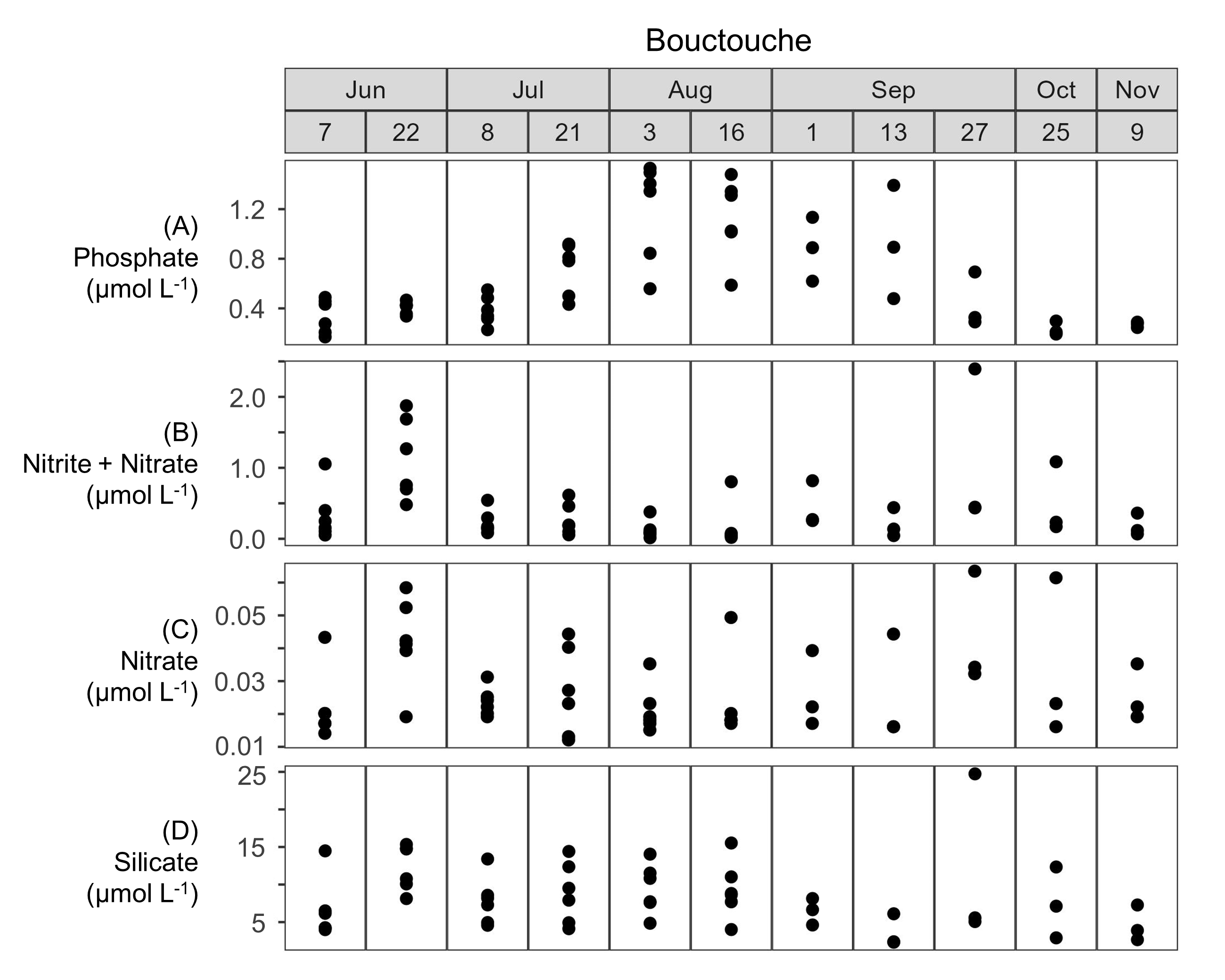

Supplement: S1 Fig — (A) Phosphate, (B) nitrite and nitrate, (C) nitrate and (D) silicate. Water samples for inorganic nutrient concentrations were collected at the same time and locations as water samples for flow cytometry, however stations are unknown here. (TIF) [file pone.0313271.s001.tif]
