# Supplementary material for: Monitoring bay-scale ecosystem changes in bivalve aquaculture embayments using flow cytometry
Source: PLoS One. 2024 Nov 5;19(11):e0313271. doi: 10.1371/journal.pone.0313271 (PMC11537403; doi:10.1371/journal.pone.0313271)
Supplement: Attachment — Submitted filename: PONE-D-24-31966.pdf [file pone.0313271.s002.pdf]

PLOS ONE

Monitoring bay-scale bivalve aquaculture ecosystem interactions using flow cytometry

--Manuscript Draft--

**Manuscript Number:**	PONE-D-24-31966
**Article Type:**	Research Article
**Full Title:**	Monitoring bay-scale bivalve aquaculture ecosystem interactions using flow cytometry
**Short Title:**	Monitoring bay-scale bivalve aquaculture ecosystem interactions using flow cytometry
**Corresponding Author:**	Hannah Sharpe<br>Fisheries and Oceans Canada Maritimes Region<br>St. Andrews, New Brunsick, CANADA
**Keywords:**	Phytoplankton; Bacteria; Eastern oyster; Shellfish; Coastal embayment
**Abstract:**	Bay-scale empirical demonstrations of how bivalve aquaculture alters plankton composition, and subsequently ecological functioning and higher trophic levels, are lacking. Temporal, inter- and within-bay variation in hydrodynamic, environmental, and aquaculture pressure limit efficient plankton monitoring design to detect bay-scale changes and inform aquaculture ecosystem interactions. Here, we used flow cytometry to investigate spatio-temporal variations in bacteria and phytoplankton (< 20 μm) composition in four bivalve aquaculture embayments. We observed higher abundances of bacteria and phytoplankton in shallow embayments that experienced greater freshwater and nutrient inputs. Depleted nutrient conditions may have led to the dominance of picophytoplankton cells, which showed strong within-bay variation as a function of riverine vs freshwater influence and nutrient availability. Although environmental forcings appeared to be a strong driver of spatio-temporal trends, results showed that bivalve aquaculture may reduce near-lease phytoplankton abundance and favor bacterial growth. We discuss aquaculture pathways of effects such as grazing, benthic-pelagic coupling processes, and microbial biogeochemical cycling. Conclusions provide guidance on optimal sampling considerations using flow cytometry in aquaculture sites based on embayment geomorphology and hydrodynamics.
**Order of Authors:**	Hannah Sharpe
	Thomas Guyondet
	Jeffrey Barrell
	Claude Belzile
	Christopher W. McKindsey
	Flora Salvo
	Anaïs Lacoursière-Roussel
**Additional Information:**	
**Question**	**Response**
**Financial Disclosure**<br><br>Enter a financial disclosure statement that describes the sources of funding for the work included in this submission. Review the submission guidelines for detailed requirements. View published research articles from *PLOS ONE* for specific examples.	Yes

Powered by Editorial Manager® and ProduXion Manager® from Aries Systems Corporation

This statement is required for submission and **will appear in the published article** if the submission is accepted. Please make sure it is accurate.<br><br>**Funded studies**<br>Enter a statement with the following details:<br>• Initials of the authors who received each award<br>• Grant numbers awarded to each author<br>• The full name of each funder<br>• URL of each funder website<br>• Did the sponsors or funders play any role in the study design, data collection and analysis, decision to publish, or preparation of the manuscript?<br><br>Did you receive funding for this work?	
Please add funding details.<br>as follow-up to "**Financial Disclosure**<br><br>Enter a financial disclosure statement that describes the sources of funding for the work included in this submission. Review the submission guidelines for detailed requirements. View published research articles from *PLOS ONE* for specific examples.<br><br>This statement is required for submission and **will appear in the published article** if the submission is accepted. Please make sure it is accurate.<br><br>**Funded studies**<br>Enter a statement with the following details:<br>• Initials of the authors who received each award<br>• Grant numbers awarded to each author<br>• The full name of each funder<br>• URL of each funder website<br>• Did the sponsors or funders play any role in the study design, data collection and analysis, decision to publish, or preparation of the manuscript?<br><br>Did you receive funding for this work?"	This project was funded by the Fisheries and Oceans Canada Aquaculture Monitoring Program.

Please select the country of your main research funder (please select carefully as in some cases this is used in fee calculation).<br>as follow-up to "**Financial Disclosure**<br><br>Enter a financial disclosure statement that describes the sources of funding for the work included in this submission. Review the submission guidelines for detailed requirements. View published research articles from *PLOS ONE* for specific examples.<br><br>This statement is required for submission and **will appear in the published article** if the submission is accepted. Please make sure it is accurate.<br><br>**Funded studies**<br>Enter a statement with the following details:<br>• Initials of the authors who received each award<br>• Grant numbers awarded to each author<br>• The full name of each funder<br>• URL of each funder website<br>• Did the sponsors or funders play any role in the study design, data collection and analysis, decision to publish, or preparation of the manuscript?<br><br>Did you receive funding for this work?"	CANADA - CA
**Competing Interests**<br><br>Use the instructions below to enter a competing interest statement for this submission. On behalf of all authors, disclose any competing interests that could be perceived to bias this work—acknowledging all financial support and any other relevant financial or non-financial competing interests.<br><br>This statement is required for submission and **will appear in the published article** if the submission is accepted. Please make sure it is accurate and that any funding	The authors have declared that no competing interests exist.

sources listed in your Funding Information later in the submission form are also declared in your Financial Disclosure statement.<br><br>View published research articles from *PLOS ONE* for specific examples.<br><br>**NO authors have competing interests**<br><br>Enter: *The authors have declared that no competing interests exist.*<br><br>**Authors with competing interests**<br><br>Enter competing interest details beginning with this statement:<br><br>*I have read the journal's policy and the authors of this manuscript have the following competing interests: [insert competing interests here]*<br><br>* typeset	
**Ethics Statement**<br><br>Enter an ethics statement for this submission. This statement is required if the study involved:<br><br>• Human participants<br>• Human specimens or tissue<br>• Vertebrate animals or cephalopods<br>• Vertebrate embryos or tissues<br>• Field research<br><br>Write "N/A" if the submission does not require an ethics statement.<br><br>General guidance is provided below. Consult the submission guidelines for detailed instructions. **Make sure that all information entered here is included in the Methods section of the manuscript.**	N/A

**Format for specific study types**<br><br>**Human Subject Research (involving human participants and/or tissue)**<br>• Give the name of the institutional review board or ethics committee that approved the study<br>• Include the approval number and/or a statement indicating approval of this research<br>• Indicate the form of consent obtained (written/oral) or the reason that consent was not obtained (e.g. the data were analyzed anonymously)<br><br>**Animal Research (involving vertebrate animals, embryos or tissues)**<br>• Provide the name of the Institutional Animal Care and Use Committee (IACUC) or other relevant ethics board that reviewed the study protocol, and indicate whether they approved this research or granted a formal waiver of ethical approval<br>• Include an approval number if one was obtained<br>• If the study involved *non-human primates*, add *additional details* about animal welfare and steps taken to ameliorate suffering<br>• If anesthesia, euthanasia, or any kind of animal sacrifice is part of the study, include briefly which substances and/or methods were applied<br><br>**Field Research**<br><br>Include the following details if this study involves the collection of plant, animal, or other materials from a natural setting:<br>• Field permit number<br>• Name of the institution or relevant body that granted permission	
**Data Availability**<br><br>Authors are required to make all data underlying the findings described fully available, without restriction, and from the time of publication. PLOS allows rare exceptions to address legal and ethical concerns. See the PLOS Data Policy and FAQ for detailed information.	Yes - all data are fully available without restriction

A Data Availability Statement describing where the data can be found is required at submission. Your answers to this question constitute the Data Availability Statement and **will be published in the article**, if accepted.<br><br>**Important:** Stating 'data available on request from the author' is not sufficient. If your data are only available upon request, select 'No' for the first question and explain your exceptional situation in the text box.<br><br>Do the authors confirm that all data underlying the findings described in their manuscript are fully available without restriction?	
**Describe where the data may be found in full sentences. If you are copying our sample text, replace any instances of XXX with the appropriate details.**<br><br>• If the data are **held or will be held in a public repository**, include URLs, accession numbers or DOIs. If this information will only be available after acceptance, indicate this by ticking the box below. For example: *All XXX files are available from the XXX database (accession number(s) XXX, XXX.).*<br>• If the data are all contained **within the manuscript and/or Supporting Information files**, enter the following: *All relevant data are within the manuscript and its Supporting Information files.*<br>• If neither of these applies but you are able to provide **details of access elsewhere**, with or without limitations, please do so. For example:<br><br>*Data cannot be shared publicly because of [XXX]. Data are available from the XXX Institutional Data Access / Ethics Committee (contact via XXX) for researchers who meet the criteria for access to confidential data.*<br><br>*The data underlying the results presented in the study are available from (include the name of the third party*	Datasets generated for this study are available in the OBIS system (doi: in progress).

and contact information or URL).<br>• This text is appropriate if the data are owned by a third party and authors do not have permission to share the data.<br><br>* typeset	
Additional data availability information:	Tick here if the URLs/accession numbers/DOIs will be available only after acceptance of the manuscript for publication so that we can ensure their inclusion before publication.

Powered by Editorial Manager® and ProduXion Manager® from Aries Systems Corporation

Manuscript
Click here to access/download;Manuscript;Manuscript_Sharpe et al_30July2024.docx

Monitoring bay-scale bivalve aquaculture ecosystem interactions using flow cytometry

Hannah Sharpe[1*], Thomas Guyondet[2], Jeffrey Barrell[2], Claude Belzile[3], Christopher W. McKindsey[4], Flora Salvo[5], Anaïs Lacoursière-Roussel[1]*

[1]St. Andrews Biological Station, Fisheries and Oceans Canada, St. Andrews, New Brunswick, Canada

[2]Gulf Fisheries Centre, Fisheries and Oceans Canada, Moncton, New Brunswick, Canada

[3]Université du Québec à Rimouski, Institut des sciences de la mer de Rimouski, Rimouski, Québec, Canada

[4]Maurice-Lamontagne Institute, Fisheries and Oceans Canada, Mont Joli, Québec, Canada

[5]Merinov, Dartmouth, Nova Scotia, Canada

* Corresponding author
Hannah Sharpe
hsharpe@unb.ca

Anaïs Lacoursière-Roussel
Anais.Lacoursiere@dfo-mpo.gc.ca

Abstract

Bay-scale empirical demonstrations of how bivalve aquaculture alters plankton composition, and subsequently ecological functioning and higher trophic levels, are lacking. Temporal, inter- and within-bay variation in hydrodynamic, environmental, and aquaculture pressure limit efficient plankton monitoring design to detect bay-scale changes and inform aquaculture ecosystem interactions. Here, we used flow cytometry to investigate spatio-temporal variations in bacteria and phytoplankton (< 20 µm) composition in four bivalve aquaculture embayments. We observed higher abundances of bacteria and phytoplankton in shallow embayments that experienced greater freshwater and nutrient inputs. Depleted nutrient conditions may have led to the dominance of picophytoplankton cells, which showed strong within-bay variation as a function of riverine vs freshwater influence and nutrient availability. Although environmental forcings appeared to be a strong driver of spatio-temporal trends, results showed that bivalve aquaculture may reduce near-lease phytoplankton abundance and favor bacterial growth. We discuss aquaculture pathways of effects such as grazing, benthic-pelagic coupling processes, and microbial biogeochemical cycling. Conclusions provide guidance on optimal sampling considerations using flow cytometry in aquaculture sites based on embayment geomorphology and hydrodynamics.

Introduction

Canadian shellfish aquaculture (i.e., bivalve mollusc) production reached 40 358 tonnes in 2022 (https://www.dfo-mpo.gc.ca/stats/aqua/aqua22-eng.html), although there is little comprehensive understanding of holistic bay-scale ecosystem effects. Spatio-temporal variation within and

between embayments (e.g., culture density, natural plankton abundance, local hydrodynamics) add complexity to monitoring efforts, interpreting, and predicting bivalve aquaculture ecosystem impacts. In particular, interactions with pelagic resources are challenging to resolve within dynamic coastal ecosystems, particularly to differentiate aquaculture effects such as grazing, artificial structure, and nutrient cycling. As Canadian bivalve aquaculture production focuses on filter-feeders that consume plankton, these interactions are critical to understand ecological functioning in aquaculture ecosystems, with implications for aquaculture management.

Decreases in phytoplankton abundance have been observed at bivalve aquaculture embayments worldwide, e.g. between Denmark and Sweden (1), in the Mediterranean (2), and in Daya Bay, South China Sea (3). However, a reduction in phytoplankton was not observed in Grande-Entrée Lagoon, Canada (4) and Sonier et al. (5) observed higher biomass accumulation (based on chlorophyll measurements) in St. Peters Bay, a Canadian embayment with one of the highest aquaculture lease coverages in the region. Bivalves retain a variety of particles during filter-feeding, including bacteria, phytoplankton, zooplankton, and detritus. Particle selection is often preferential and influenced by particle abundance, size, shape, morphology, motility, toxicity, and nutritional content, that varies as a function of seasonality and bay dynamics (6–10). However, bivalves must first filter a sufficient quantity of particles before preferential particle selection can occur (7). They capture particles of increasing size with increasing efficiency until a given species' size threshold has been met (11). Most bivalves have a 100 % retention efficiency for particles larger than 4 µm (12–15). Sonier et al. (5) reported that cultivated blue mussels (*Mytilus edulis*) have a retention efficiency of 20 % ± 2 % for picophytoplankton (0.2 to 2 µm) and 60 % ± 3.5 % for nanophytoplankton (2 to 20 µm). The general consensus is that picoplankton are retained at an efficiency of 0 to 30 %, depending on

bivalve species and life stage (16) and that phytoplankton > 4 µm are the most efficiently retained food source for bivalves (17–20).

Despite their important role in marine food webs and nutrient cycling, it is difficult to characterize the overall contribution of heterotrophic bacteria to bivalve diets because they can be digested as free-living cells, bacterial aggregates, or attached to seston. Langdon and Newell (21) reported that the Eastern oyster *Crassostrea virginica* retained free-floating (unattached) bacteria with 5.0 % efficiency, while the mussel *Geukensia demissa* retained bacteria with 15.8 % efficiency. Several authors have suggested that 20 to 30 % of bacteria exist within aggregates exceeding 5 µm in size (22,23), which would be expected to be retained with 100 % efficiency. Aggregates are formed when unicellular and multicellular organisms produce exopolymers that coalesce to form gels (24). Both oysters and mussels can enhance the concentration of transparent exopolymer particles in the environment, thereby promoting aggregate formation (25,26). Due to their carbon to nitrogen ratios (3.5 and 6.6 for bacteria and phytoplankton, respectively), it is thought that although bacteria may not contribute much to the metabolic carbon requirements of bivalves, they may provide substantially to bivalve nitrogen requirements (21,27,28).

Flow cytometry is a rapid (< 3 min per sample), highly sensitive and low-cost method to analyze organisms < 20 µm (29,30) and has been a globally-useful technology for aquatic research and marine monitoring for decades (31). The benefits of using flow cytometry to quantify changes in bacteria and phytoplankton, and consequently track changes in aquatic environments. have been well-documented for coastal ecosystems (30,32–34). The method has been used to characterize ecosystem dynamics in bivalve aquaculture sites worldwide, with results reported as absolute abundance of cells (3,35), biomass estimates (1,36), or both (37,38).

While abundance and relative abundance informs community structure and biodiversity, and shows better correlation to variations in environmental conditions (39,40), a limit of flow cytometry is the ability to accurately assess the biovolume, and therefore biomass, of the cells analyzed. This has important implications for ecosystem productivity and food availability to higher trophic levels, and must therefore be measured using additional methods such as bulk filtrations, microscopy counts, or imaging technology.

Flow cytometry can be used to distinguish two physiologically and ecologically distinct bacterial groups: cells with low nucleic acid content (LNA) and cells with high nucleic acid content (HNA) (41–45). LNA bacterial cells have streamlined and small genomes, resulting in lower metabolic rates and less capacity to utilize dissolved organic carbon, and thus live in specific ecological niches (46,47). Due to their compact genomes, they thrive in nutrient-limited environments whereas HNA cells thrive in environments with high levels of inorganic nutrients and organic matter (48–51). The relative abundance of HNA to LNA is therefore an informative indicator of environmental conditions and nutrient availability (52).

Flow cytometry is also used to distinguish between pico- and nano-phytoplankton (0.2 to 2 µm and 2 to 20 µm, respectively) in addition to two broad phytoplankton communities: cyanobacteria and eukaryotes. Different cyanobacteria species contain light-harvesting pigments in the phycobiliprotein family such as phycocyanin (PC) and phycoerythrin (PE) in differing quantities and are identified using flow cytometry (53). PC is an indicator for freshwater cyanobacteria (34) while PE is present in high quantities in marine cyanobacterium *Prochlorococcus* and *Synechococcus,* the two most abundant oxygenic phototrophs on Earth (54). Eukaryotes within both the pico and nano size fractions are principally members of the

classes Chlorophyceae (green algae), Pelagophyceae (heterokont algae), Dinophyceae (dinoflagellates), and Bacillariophyceae (diatoms).

Experimental studies have shown that plankton depletion by mussel grazing negatively impacts the fitness of important Canadian fisheries resources, such as lobster larvae (55). However, empirical aquaculture ecosystem interactions are still not well-understood. The present study investigates spatio-temporal variations in bacteria and phytoplankton (< 20 µm) measured using flow cytometry in four Atlantic Canadian bivalve aquaculture embayments. Here, we evaluate 1) inter-bay comparisons of late-summer surface bacteria and phytoplankton composition and 2) within-bay spatio-temporal variations in plankton composition at two deep embayments in Nova Scotia and two shallow embayments in New Brunswick. This work is part of the Aquaculture Monitoring Program, launched by Fisheries and Oceans Canada, which helps inform policy and decision-making through assessments of the impacts of shellfish (bivalve molluscs) aquaculture on marine ecosystems.

Methods

Water samples were collected from four Eastern oyster (*Crassostrea virginica*) floating cage aquaculture embayments in Atlantic Canada (Fig 1, Table 1). The embayments were selected to span a range of oceanographic conditions and aquaculture lease coverage, with varying pre-existing knowledge of the hydrodynamics and bivalve aquaculture-environment interactions in each of them. Despite regional differences in geomorphology and hydrodynamics, these embayments have been shown to be ecologically similar at the mesozooplankton level (56). The quantity of bivalves per lease is often unknown as this is generally proprietary information. Within each embayment, samples were collected at a minimum of three stations to gain a general

understanding of spatial dynamics for bacteria and phytoplankton abundance and composition (Fig 1). The deep Nova Scotia embayments (Country Harbour, Whitehead) had a single point of exchange with the open ocean that followed a linear path and stations were labeled as Inner, Mid, and Outer. The shallow New Brunswick embayments (Bouctouche, Cocagne) had a more circular coastline and sampling was conducted in a grid format such that stations were labeled according to geographic location and cardinal directions (South, Central, North). At Bouctouche, additional sampling was done outside the embayment (Out station) and in the river (River station). All station locations were chosen to sample areas both near and far from leases while keeping an approximately even spacing of stations within embayments.

Fig 1. Location of sampling stations within four bivalve aquaculture embayments in Atlantic Canada. (A-B) Location of embayments in New Brunswick and Nova Scotia in Atlantic Canada. (C-F) Location of sampling stations in relation to the bivalve aquaculture leases. Red indicates active leases and pink indicates empty leases at time of sampling.

Table 1. Data summary table for bacteria and phytoplankton sampling (0.2 to 20 µm).

Embayment	Bay area (km^2)	Lease area (km^2)	Cover (%)	Watershed area (km^2)	Tidal range (m)	Max depth (m)	Sampling dates	n
Country Harbour	10.4	0.84 (empty during sampling)	0.0 during sampling	235.6	0.53-1.87 (Isaacs Harbour, 00535)	21.9	Aug 24 (2021)	18
Whitehead	1.7	0.23	13.9	63.1	0.45-1.80 (Whitehead, 00545)	14.3	Aug 25 (2021)	27

Bouctouche	28.4	1.45	5.1	890.0	0.23-1.28 (St. Thomas De Kent, 01815)	10.0	May 25; Jun 7, 22; Jul 8, 21; Aug 3, 16; Sep 1, 13, 27; Oct 25; Nov 9 (2022)	72
Cocagne	18.9	1.44	7.6	355.4	0.32-1.05 (Cocagne, 01812)	8.3	Jun 24, Jul 8, Aug 4, Sep 8 (2021)	40

Cover (%) represents the ratio of the lease area to the bay area. Watershed area is retrieved from New Brunswick Department of Natural Resources and Energy Development (https://hub.arcgis.com/datasets/19d7ac97045442fbac2b0f80d66c53ec_0/about) and the Government of Nova Scotia (https://www.arcgis.com/home/item.html?id=b283921b14214a3889cb47fe0e0aab61). The tidal range (m) is presented as the range between the lower low water mean tide to higher high water mean tide, from the nearest Canadian Hydrographic Service tide station to the sampling stations (https://wla.iwls.azure.cloud.dfo-mpo.gc.ca/stationMgmt). n refers to the number of samples obtained at each embayment.

Embayment descriptions and sampling design

Country Harbour (10.4 km^2) and Whitehead (1.7 km^2) are long, narrow channels on the eastern shore of Nova Scotia, Canada (Fig 1, Table 1). These embayments are relatively deep (all stations have depths $<$ 16.3 m) with a semidiurnal tidal influence. Although blue mussels have historically been cultured, Eastern oysters are currently the main cultured bivalve at these embayments, covering 13.9 % of the bay area at Whitehead, where 27 samples were collected on 25 August 2021, at three tide phases (high, mid-falling, low) and from three water depths. All leases were empty during sampling at Country Harbour, where 18 samples were collected on 24 August 2021 at two tide phases (high, low) and from three water depths.

Bouctouche (28.4 km^2) and Cocagne (18.9 km^2) are large enclosed bays on the eastern coast of New Brunswick, Canada (Fig 1, Table 1). All stations have depths < 4 m, therefore all samples were collected from the surface (1 m below surface). Tidal influence is not considered to be strong in these embayments and was thus not evaluated as a factor. Bouctouche and Cocagne consist of numerous floating cage Eastern oyster leases, covering 5.1 % and 7.6 % of the bay areas, respectively. Sampling at Bouctouche was conducted biweekly from May to November 2022, with water collected from four locations within the embayment, one outside the embayment, and one at the mouth of the river, for a total of 72 samples. Sampling at Cocagne was conducted monthly from June to September 2021 at 10 locations within the embayment, for a total of 40 samples.

Sample collection

Water samples were collected using a 5 L Niskin bottle and, immediately upon collection, 4.5 mL was combined with 20 µL Grade 1 glutaraldehyde (Sigma; 0.1 % final concentration) in cryovials and kept at ambient temperature in the dark for 15 minutes. Subsamples were then placed on ice while in the field and subsequently stored at -80 °C until analysis at Institut des Sciences de la Mer de l'Université du Québec à Rimouski (Canada). At Country Harbour and Whitehead, samples were collected at three depths: surface (S), mid (M), and deep (D). Surface samples were collected 1 m below surface, mid samples were collected approximately halfway down the water column, and deep samples were collected 4 m above seafloor. At Bouctouche and Cocagne, only surface samples were collected due to the shallow water column.

Flow cytometry

Subsamples for heterotrophic bacteria enumeration were stained with SYBR Green I (Invitrogen) following Belzile et al. (57). Bacteria (and archaea, collectively referred to here as bacteria) were counted with a CytoFLEX flow cytometer (Beckman Coulter) using the blue laser (488 nm). Green fluorescence of nucleic acid-bound SYBR Green I was measured at 525 nm (525/40 nm BP). The cytograms obtained were analyzed using CytExpert v2.3 software and the same regions of the side scatter vs. green fluorescence plots were ascribed to LNA and HNA bacterial cells.

Subsamples for < 20 µm autotroph abundances (i.e. PE-cyanobacteria, PC-cyanobacteria and autotrophic eukaryotes, collectively referred to as phytoplankton) were analyzed using a CytoFLEX flow cytometer (Beckman Coulter) following Araújo et al. (58) equipped with blue (488 nm) and red (638 nm) lasers. Using the blue laser, forward scatter, side scatter, orange fluorescence from PE (582/42 nm BP) and red fluorescence from chlorophyll (690/50 nm BP) were measured. The red laser was used to excite the red fluorescence of PC (660/20 nm BP). Two µm diameter polystyrene microspheres (Fluoresbrite YG, Polysciences) were added to each sample as an internal standard. Pico- (0.2 to 2 µm) and nano-autotrophs (2 to 20 µm) were discriminated based on a forward scatter calibration using algal cultures. Nano-sized autotrophs containing PE or PC were ascribed to nano-cyanobacteria, but may also have been cryptophytes or rhodophytes (59).

Seston and chlorophyll *a*

At Bouctouche and Cocagne, additional water was collected in conjunction with that used for flow cytometry to allow the analysis of suspended particulate matter (SPM), particulate organic matter (POM), and chlorophyll *a* (chl *a*). For SPM measurements, 1 L duplicates were filtered onto 47 mm Whatman GF/C pre-combusted (500 °C for 4 h in a muffle furnace) and pre-

weighed filters (nominal porosity of 1.2 µm) and rinsed with of 0.5 M ammonium formate. The filters were subsequently dried at 70 °C for 24 h, cooled in a desiccator for 2 h, and reweighed for the determination of SPM. Organic matter was then removed by combusting the dried filters at 500 °C for 4 h, then the filters were cooled in a desiccator for 2 h and reweighed for the determination of POM. For chl *a* measurements, duplicate subsamples of 20 and 250 mL were filtered onto 25 mm polycarbonate track-etched (PCTE) filters with nominal porosities of 0.2 and 3.0 µm, respectively. The filters were frozen at -20 °C in the dark until analysis. The pigments retained on the filters were extracted in 10 mL of 90 % ice cold acetone and stored at -20 °C in the dark for 24 h. The samples were then warmed to room temperature and the supernatant transferred to a borosilicate tube for fluorescence measurements using the modified fluorometric methods (i.e., narrow bandpass filters to reduce interference from phaeopigments) without acidification (60). Samples were analyzed using a Turner Trilogy fluorometer calibrated using chl *a* standards from Turner (SKU: 10-850).

Statistical analysis

Two-dimensional non-metric multidimensional scaling (NMDS) ordinations (61) were used to graphically display spatio-temporal patterns in bacteria and phytoplankton community composition (taxa and abundance). Permutational multivariate analysis of variance (PERMANOVA) was used (number of permutations = 9999) to evaluate variation in plankton communities over time. NMDS ordinations and PERMANOVAs were constructed using a Bray-Curtis dissimilarity matrix of square root transformed abundance data. All analyses were done using the *vegan* package in *R* (62); the *metaMDS* function was used for NMDS ordinations, and the *adonis2* function was used for PERMANOVAs. Boxplots were used to visualize spatio-temporal patterns in bacteria and phytoplankton composition, showing the first, second, and third

quartiles, and lines extending from the boxes indicate the minimum and maximum values up to 1.5 times the interquartile range. The data was not normally distributed therefore the Mann-Whitney U test, a nonparametric test, was used to determine significantly different groups within all factors.

For inter-bay comparisons, surface samples collected from August 4 to September 8 (referred to throughout as late-summer samples) at all four embayments were used. Within-bay statistical analysis conducted for the deep Nova Scotia embayments (Country Harbour, Whitehead) considered the effect of water depth (S, M, D), tide phase (low, high), station (Inner, Mid, Outer), and their interactions as sources of variance (factors) in bacteria and phytoplankton assemblage composition. When testing differences in tide phase, mid-falling samples were removed to only test low and high tides. Within-bay statistical analysis conducted for the shallow New Brunswick embayments (Bouctouche, Cocagne) considered month (Jun, Jul, Aug, Sep), station (South, Central, North), and their interactions as sources of variance (factors) in bacteria and phytoplankton assemblage composition. For Bouctouche, only stations within the embayment (Central and North; 2 locations sampled within each station over 7 dates making a total of 14 samples per station) from June 22 to September 13 were used to provide consistency with Cocagne and allow data comparison.

Results

Inter-bay comparisons

Late-summer bacteria and phytoplankton composition in surface waters varied significantly between embayments (Fig 2, Table 2), which showed differences primarily between the shallow and deeper embayments. The deeper embayments showed no overlap and Country Harbour showed two distinct phytoplankton groups based on riverine (Inner station at high and low tide,

as well as Mid station at low tide) and marine (Outer station at high and low tide, as well as Mid station at high tide) influence. The shallow embayments showed similar phytoplankton composition between the Cocagne stations in August and the Bouctouche stations in September.

Fig 2. Two-dimensional non-metric multidimensional scaling ordination (NMDS) showing variation in bacteria and phytoplankton (0.2 to 20 µm) composition of late-summer surface samples. Color represents embayment while shape represents month. For Country Harbour, groupings based on riverine (R) vs marine (M) influence are highlighted.

Table 2. Summary of statistically significant permutational multivariate analysis of variance (PERMANOVA) results based on a Bray-Curtis dissimilarity matrix of square root transformed bacteria and phytoplankton (0.2 to 20 µm) abundances.

		Bacteria				Phytoplankton			
	df	SS	R^2	F	p	SS	R^2	F	p
**Inter-bay comparisons**									
**Embayment**	**3**	**0.298**	**0.789**	**51.413**	**<0.001**	**0.936**	**0.630**	**22.167**	**<0.001**
Residuals	39	0.075	0.202			0.367	0.367		
Total	42	0.373	1.000			1.000	1.000		
**Country Harbour**									
**Station**	**2**	**0.029**	**0.517**	**10.510**	**0.002**	**0.084**	**0.507**	**11.193**	**0.001**
Tide	1	0.004	0.076	3.098	0.097	0.010	0.062	2.739	0.113
**Station*Tide**	2	0.006	0.111	2.260	0.145	**0.027**	**0.159**	**3.521**	**0.052**
Residual	12	0.017	0.295			0.045	0.272		
Total	17	0.056	1.000			0.167	1.000		
**Whitehead**									

**Station**	**2**	**0.023**	**0.314**	**31.973**	**<0.001**	**0.013**	**0.113**	**6.282**	**0.002**
**Water sample depth**	**2**	**0.030**	**0.405**	**41.177**	**<0.001**	**0.065**	**0.542**	**30.197**	**<0.001**
**Station* Water sample depth**	**4**	**0.014**	**0.193**	**9.805**	**<0.001**	**0.022**	**0.183**	**5.109**	**<0.001**
Residual	18	0.006	0.088			0.019	0.162		
Total	26	0.073	1.000			0.119	1.000		
**Bouctouche**									
**Month**	**3**	**0.046**	**0.670**	**27.451**	**<0.001**	**0.250**	**0.728**	**21.099**	**<0.001**
**Station**	**1**	**0.010**	**0.146**	**17.968**	**<0.001**	0.004	0.011	0.967	0.386
Month*Station	3	0.001	0.020	0.839	0.492	0.011	0.031	0.911	0.502
Residual	20	0.011	0.163			0.079	0.230		
Total	27	0.069	1.000			0.344	1.000		
**Cocagne**									
**Month**	**3**	**0.019**	**0.175**	**8.296**	**<0.001**	**0.421**	**0.734**	**69.178**	**<0.001**
**Station**	**2**	**0.056**	**0.512**	**36.475**	**<0.001**	**0.024**	**0.042**	**5.953**	**<0.001**
**Month*Station**	**6**	**0.013**	**0.117**	**2.779**	**0.025**	**0.070**	**0.123**	**5.793**	**<0.001**
Residual	28	0.022	0.196			0.568	0.099		
Total	39	0.110	1.000			0.572	1.000		

df: degrees of freedom; SS: sum of squares; R^2: coefficient of variation; F: F statistic by permutation, p: significance by 9999 permutations. Significant effects are shown in bold $p < 0.05$).

Late-summer bacteria abundance differed significantly between all four embayments, with the highest and lowest abundances at Bouctouche and Country Harbour, respectively (Fig 3). The relative abundance of HNA bacteria was greater in the shallow New Brunswick embayments than the deeper Nova Scotia embayments. Phytoplankton abundance was

significantly higher at Bouctouche relative to the other three embayments, which were statistically similar. Country Harbour had the highest proportion of picophytoplankton and cyanobacteria and, within both the shallow and deeper sets of embayments, relative abundances were lower at the embayments with higher lease coverage.

Fig 3. Boxplots for flow cytometry data (0.2 to 20 µm) of late-summer surface samples from Bouctouche (Bou), Cocagne (Coc), Country Harbour (CH) and Whitehead (WH). (A) Bacteria abundance, (B) relative abundance of bacteria with high nucleic acid content (HNA) in relation to all bacteria, (C) phytoplankton abundance, (D) relative abundance of picophytoplankton in relation to all phytoplankton cells, (E) relative abundance of cyanobacteria in relation to all phytoplankton cells. Embayments that differ significantly, based on the Mann Whitney U tests, are represented by red boxes.

Spatial and tidal effects at deeper embayments

At Country Harbour, bacteria abundance ranged from 1.4×10^6 to 2.7×10^6 cells mL^{-1} and phytoplankton abundance ranged from 0.9×10^5 to 2.5×10^5 cells mL^{-1} (Fig 4). HNA represented 42.3 to 55.8 % of total bacteria, while picophytoplankton and cyanobacteria ranged from 90.7 to 94.9 % and 74.1 to 86.8 % of total autotrophic cells, respectively. Station had a significant effect on bacteria and phytoplankton composition (Table 2), with abundances decreasing from Inner to Outer stations while the relative abundance of picophytoplankton increased from the Inner to Outer stations, all of which were not significantly influenced by tide phase or water sample depth (Figs 4-6). Tide phase did, however, significantly impact the relative abundances of HNA (p = 0.05) and cyanobacteria (p = 0.03), with greater values during low tide (average 50.9 % HNA at low tide, 46.7 % HNA at high tide; Fig 6). As well, overall greater proportions of HNA were

recorded at the Inner (average 50.3 %) and Mid (average 49.6 %) stations compared to the Outer station (average 46.5%), indicating that greater riverine influence increases HNA cells (Fig 6). None of the parameters measured varied as a function of water sample depth.

Fig 4. Flow cytometry data (0.2 to 20 µm) from Country Harbour and Whitehead. (A) Bacteria abundance, (B) relative abundance of bacteria with high nucleic acid content (HNA) in relation to all bacteria, (C) phytoplankton abundance, (D) relative abundance of picophytoplankton (PPP) and cyanobacteria in relation to all phytoplankton cells, and (E) relative abundance of pico- (0.2 to 2 µm) and nano- (2 to 20 µm) sized eukaryotes, PC-cyanobacteria, and PE-cyanobacteria. For each embayment, samples are grouped by station and water sample depth (S = surface, M = mid, D = deep).

Fig 5. Two-dimensional non-metric multidimensional scaling ordination (NMDS) showing variation in bacteria and phytoplankton (0.2 to 20 µm) composition from Country Harbour and Whitehead. Colors represent embayment and tide phase and shape represents station. Water sample depth (S = surface, M = mid, D = deep) is indicated by text.

Fig 6. Boxplots for flow cytometry data (0.2 to 20 µm) from Country Harbour and Whitehead. (A) Bacteria abundance, (B) relative abundance of bacteria with high nucleic acid content (HNA) in relation to all bacteria, (C) phytoplankton abundance, (D) relative abundance of picophytoplankton in relation to all phytoplankton cells, (E) relative abundance of cyanobacteria in relation to all phytoplankton cells. For each embayment, boxplots show data distribution between tide phase (M-F = mid-falling), station, and water sample depth (S = surface, M = mid, D = deep). Red brackets highlight significantly different groups based on the Mann Whitney U test.

At Whitehead, bacteria abundance ranged from 1.6×10^6 to 3.4×10^6 cells mL^{-1} and phytoplankton abundance ranged from 0.8×10^5 to 1.7×10^5 cells mL^{-1} (Fig 4). HNA represented 32.9 to 55.1 % of total bacteria, while the relative abundances of picophytoplankton and cyanobacteria ranged from 70.1 to 87.0 % and 55.3 to 80.6 %, respectively. Station and water sample depth both had a significant influence on bacteria and phytoplankton composition (Table 2). The Inner and Mid stations were similar for all measured parameters, whereas the Outer station had significantly lower bacteria abundance and higher relative abundances of picophytoplankton, and cyanobacteria. Stations did not differ significantly for phytoplankton abundance or the relative abundance of HNA. Surface samples had significantly higher bacteria abundance, a higher proportion of HNA bacteria, and significantly lower relative abundances of picophytoplankton and cyanobacteria, compared to mid and deep samples (Figs 4-6). Phytoplankton abundance was similar between surface and mid samples but significantly lower in the deeper samples. Tide phase did not significantly influence any of the parameters measured.

Spatial and seasonal effects at shallow embayments

At Bouctouche, total bacteria abundance ranged from 2.0×10^6 to 7.7×10^6 cells mL^{-1} and phytoplankton abundance ranged from 0.4×10^5 to 6.3×10^5 cells mL^{-1} (Fig 7). Abundances of picoPC- and picoPE-cyanobacteria were highest at the River and Out stations, respectively, while abundances of picoeukaryotes were highest within the embayment (Fig 8). Month and station both had a significant effect on bacteria composition, whereas only month significantly affected phytoplankton composition (Table 2). Phytoplankton showed a stronger seasonal trend (Fig 9) with peak abundance in August, while bacteria abundance was highest in both August and September (Fig 10). The proportion of HNA bacteria ranged from 46.6 to 60.0 % and showed high spatio-temporal variability with values > 58 % recorded in May (North, River,

Central stations), June (River station), August (North, Central, Out stations) and September (North station). Overall, the relative abundance of picophytoplankton ranged from 57.0 to 93.7 % and the relative abundance of cyanobacteria ranged from 17.0 to 86.9 %. Phytoplankton composition showed a strong seasonal influence, with assemblages dominated by pico- and nano-eukaryotes from May 25 to July 7, by picoPC-cyanobacteria from June 22 to September 13, then by picoPE-cyanobacteria and pico- and nano- eukaryotes from September 27 to November 9. The relative abundance of cyanobacteria was greatest at the River station from May to September, when picoPC-cyanobacteria comprised the majority of phytoplankton cells. Stations within the embayment (Central and North) did not differ significantly for any of the parameters measured (Fig 10).

Fig 7. Flow cytometry data (0.2 to 20 µm) from Bouctouche and Cocagne. (A) Bacteria abundance, (B) relative abundance of bacteria with high nucleic acid content in relation to all bacteria, (C) phytoplankton abundance, (D) relative abundance of picophytoplankton (PPP) and cyanobacteria in relation to all phytoplankton cells, and (E) relative abundance of pico- (0.2 to 2 µm) and nano- (2 to 20 µm) sized eukaryotes, PC-cyanobacteria, and PE-cyanobacteria.

Fig 8. Flow cytometry picophytoplankton abundance data from Bouctouche and Cocagne. (A) PicoPC-cyanobacteria, (B) picoPE-cyanobacteria and (C) picoeukaryotes. Colors represent stations.

Fig 9. Two-dimensional non-metric multidimensional scaling ordination (NMDS) showing variation in bacteria and phytoplankton (0.2 to 20 µm) composition from June 22 to September 13 from Bouctouche and Cocagne. Colors represent embayment and sampling month and shape represents station.

Fig 10. Boxplots for flow cytometry data (0.2 to 20 μm) from Bouctouche and Cocagne. (A) Bacteria abundance, (B) relative abundance of bacteria with high nucleic acid content (HNA) in relation to all bacteria, (C) phytoplankton abundance, (D) relative abundance of picophytoplankton in relation to all phytoplankton cells, (E) relative abundance of cyanobacteria in relation to all phytoplankton cells. For each embayment, boxplots show data distribution between months and stations (S = South, C = Central, N = North). Red brackets highlight significantly different pairwise comparisons between stations based on the Mann Whitney U tests. Significantly different months for Bouctouche (below diagonal) and Cocagne (above diagonal) are represented using red boxes.

At Cocagne, bacteria abundance ranged from 2.8×10^{6} to 6.9×10^{6} cells mL^{-1} and phytoplankton abundance ranged from 0.8×10^{5} to 3.5×10^{5} cells mL^{-1} (Fig 7). Abundances of picoPC- and picoPE-cyanobacteria were generally higher near the river and outer embayment, respectively, while abundances of picoeukaryotes were highest at the North station in August (Fig 8). Month, station, and their two-way interaction significantly affected bacteria and phytoplankton composition (Table 2). Both groups showed increasing abundance from June to August, followed by a decrease in September although the seasonal pattern was less pronounced at Cocagne compared to Bouctouche (Figs 7-10). HNA bacteria ranged from 51.7 to 68.4 % and consistently decreased from June to September, with the highest proportion of HNA observed at the North station (68.4 % in June, 57.9 % in September) or near-river at the South station (65.6 % in July, 61.0 % in August). Phytoplankton composition showed a strong seasonal influence with the relative abundance of cyanobacteria (21.3 to 64.6 %) peaking in July and the relative abundance of picophytoplankton (63.4 to 92.9 %) peaking in August. Samples were generally

similar between stations, with the exception of bacteria abundance, which was significantly greater at the North station (Fig 10).

Bulk organic matter and chl *a* at shallow embayments

At Bouctouche, SPM ranged from 3.5 to 19.7 mg L^{-1} and POM ranged from 2.0 to 7.3 mg L^{-1} (therefore, 26.0 to 60.2 % organics, Fig 11). Total chl *a* ranged from 1.3 to 7.2 µg L^{-1} with the relative concentration of the pico size fraction (0.2 to 3 µm) ranging 27.2 to 88.6 %. Stations within the embayment (Central and North) did not differ significantly for any of the parameters measured (Fig 12). The months of June to September were generally statistically similar for all parameters measured (Fig 12) with the exception of June and July (relative contribution of pico-sized chl *a*), July and September (SPM, POM), and August and September (relative concentration of organics).

Fig 11. Bulk organic matter and chl *a* from Bouctouche and Cocagne. (A) Suspended particulate matter (SPM), (B) particulate organic matter (POM), (C) relative percentage of POM to all SPM, (D) total chl *a* and (E) relative abundance of chl *a* 0.2 to 3 µm (of total chl *a*).

Fig 12. Boxplots of bulk organic matter and chl *a* from Bouctouche and Cocagne. (A) Suspended particulate matter (SPM), (B) particulate organic matter (POM), (C) relative percentage of POM to all SPM, (D) total chl *a* and (E) relative abundance of chl *a* 0.2 to 3 µm (of total chl *a*). For each embayment, boxplots show data distribution between months and stations (S = South, C = Central, N = North). Red brackets highlight significantly different pairwise comparisons between stations based on the Mann Whitney U tests. Significantly different months for Bouctouche (below diagonal) and Cocagne (above diagonal) are represented using red boxes.

At Cocagne, SPM ranged from 3.0 to 17.5 mg L^{-1} and POM ranged from 2.3 to 5.9 mg L^{-1} (therefore, 33.9 to 76.4 % organic, Fig 11). Total chl *a* ranged from 1.3 to 8.6 µg L^{-1} with the relative concentration of the pico size fraction (< 3 µm) ranging 33.3 to 83.0 %. The North station had statistically higher concentrations of SPM, POM, and chl *a*, as well as lower concentrations of pico-sized chl *a* (Fig 12). June had the highest concentrations of SPM and POM, while August had the highest percentage of organics.

Linear regressions (Fig 13) showed that POM concentrations were correlated to flow cytometry determined plankton ($R^2 = 0.11$, $p = 0.04$) and bacteria ($R^2 = 0.11$, $p = 0.03$) abundance at Cocagne, whereas it was only correlated to phytoplankton abundance ($R^2 = 0.07$, $p = 0.03$) at Bouctouche, although all correlations were generally weak. Correlations between chl *a* and POM concentrations ($R^2 = 0.06$, $p = 0.04$) , as well as the relative contribution of picophytoplankton measured from chl *a* filtrations and cell abundance ($R^2 = 0.34$, $p < 0.001$) were observed at Cocagne but not Bouctouche.

Fig 13. Linear regressions comparing abundance and biomass data from Bouctouche and Cocagne. (A) particulate organic matter (POM) vs total plankton (0.2 to 20 µm) abundance, (B) POM vs bacteria abundance, (C) POM vs phytoplankton abundance, (D) total chl *a* vs POM, (E) total chl *a* vs phytoplankton abundance, and (F) relative biomass of chl *a* 0.2 to 3 µm (of total chl *a*) vs picophytoplankton relative abundance. Only the R^2 and p values of statistically significant relationships are shown.

Discussion

Bacteria and phytoplankton form the base of marine food webs and are the primary food source for larger nano- and micro-grazers (e.g., heterotrophic flagellates, ciliates, copepods) that are in

turn grazed by larger zooplankton. Changes in bacteria and phytoplankton can therefore lead to repercussions in the marine food chain, with impacts on metazooplankton and fish (63–65). For example, experimental studies have shown that plankton depletion by mussel grazing may negatively impact the fitness of important Canadian fisheries resources, such as lobster (55). However, these dynamics have not been observed and quantified empirically due to the complexity and dynamics of these ecosystems.

Here, inter- and within-bay patterns in bacteria and phytoplankton composition were observed in conjunction with bay dynamics and bivalve aquaculture. Higher abundances of bacteria were observed in shallow enclosed embayments that experienced greater freshwater and nutrient input. Seasonal patterns in phytoplankton abundance and composition were recorded at both New Brunswick embayments, with stronger seasonal variation observed in 2022 during record high sea surface temperatures and precipitation levels. Depleted nutrient conditions during summer months may have led to the dominance of picophytoplankton abundance and biomass. Picophytoplankton functional groups showed clear spatio-temporal trends, with greatest PC-cyanobacteria (absolute and relative) abundance associated with freshwater influence (River station) whereas PE-cyanobacteria abundance was associated with marine influence (Out station). Absolute picoeukaryote abundance was greatest within the embayment (North, Central and South stations) and varied as a function of seasonality, with particularly clear station-driven differences in August. Although environmental forcings appeared to be a strong driver of spatio-temporal trends, results showed that bivalve aquaculture may reduce near-lease phytoplankton abundance and favor bacterial growth.

Inter-bay dynamics

Bacteria and phytoplankton composition in surface waters differed significantly between embayments, showing similarities between the two shallow, enclosed New Brunswick embayments (Bouctouche, Cocagne), and the two deeper, channel-shaped Nova Scotia embayments (Country Harbour, Whitehead). The shallow enclosed embayments generally had higher bacteria abundance and relative abundance of HNA bacterial cells. HNA cells thrive in waters with high levels of inorganic nutrients and organic matter (48–51), suggesting that the shallow embayments had higher nutrient availability. Phytoplankton abundance was significantly greater at Bouctouche than the other three embayments, which may be driven by nutrient inputs from the watershed area, which is much greater at Bouctouche (890 km^2) than the others (355.4, 235.6 and 63.1 km^2 for Cocagne, Country Harbour and Whitehead, respectively). Agricultural land use in the New Brunswick watersheds (12 and 8 % for Bouctouche and Cocagne, respectively; DFO unpublished data) also likely contributed more nutrients than in Nova Scotia embayments, < 1 % in Country Harbour and Whitehead (66). The shallow embayments may also be more susceptible to resuspension of seafloor sediments during high-wind events, which may re-introduce nutrients to the water column. Additionally, a difference in oceanic influence may create differences, as the deeper Nova Scotia embayments open to the Atlantic Ocean, whereas the shallow New Brunswick embayments open to the Northumberland Strait, which is a somewhat enclosed, shallow coastal area in the Gulf of St. Lawrence, influencing water temperatures which are warmer in the Northumberland Strait than the open Atlantic Ocean. A difference in water sources was also observed at Country Harbour (Fig 2B), where different phytoplankton composition was measured between river influenced samples (inner station at high and low tide as well as mid station at low tide) and marine influenced samples (outer station at high and low tide as well as mid station at high tide).

Seasonal effects

Temperate bivalve aquaculture embayments are influenced by strong seasonality and variation in marine environmental conditions (e.g., water temperature, terrestrial freshwater and nutrient input, sea ice cover, water column stratification, light availability). Previous studies have shown that bay-specific characteristics such as local hydrodynamics drive environmental factors for bacteria and phytoplankton abundances. For example, seasonal plankton succession in a deep (20 to 50 m) Canadian bivalve aquaculture embayment (South Arm) on the northeast coast of Newfoundland is driven by water column stratification (67). Timing and intensity of stratification are generally influenced by freshwater input and atmospheric forcing (i.e. air temperature and wind) and ultimately affect nutrient and light availability, which are the most important variables controlling phytoplankton growth in subarctic marine systems (68,69). In the present study, seasonality was investigated in much shallower embayments (< 10 m) and well-mixed water columns, such that stratification may not be the driving force for plankton growth. Inorganic nutrient measurements were obtained from Bouctouche (S1 Fig) in conjunction with water collection for flow cytometry. Results suggest that nitrogen availability may be the limiting factor for plankton growth at Bouctouche, as low nitrate and nitrite levels were observed during the summer, likely due to depletion by plankton uptake, whereas phosphate and silicate remained available in the water column.

Heterotrophic bacteria (here including both bacteria and archaea) and phytoplankton abundance (Figs 4 and 7) in marine environments are highly interconnected and often are mutually beneficial (70). This is because phytoplankton cell lysis and excretion are used as a source of organic matter for bacteria, which subsequently re-mineralize organic matter, providing nutrients for phytoplankton. However, both groups compete for the same pool of

nutrients and phytoplankton typically dominate when fresh supplies of nutrients are present, whereas bacteria growth often peaks following maximal phytoplankton production and dominates in oligotrophic waters (71–73). This pattern was observed in Bouctouche (sampled in 2022; Fig. 10) whereas bacteria abundance peaked first, followed by phytoplankton abundance in Cocagne and South Arm (both embayments sampled in 2021). Overall, the timing in peak phytoplankton abundance was shifted later by a month at South Arm (September), compared to Bouctouche and Cocagne (August), likely due to differences in latitude, water column depth and stratification, and oceanic influence (i.e., Newfoundland shelf vs Northumberland Strait in southern Gulf of Saint- Lawrence).

In addition to intrinsic differences in hydrodynamics and river influence, differences in the timing, magnitude, and plankton composition between Cocagne and Bouctouche may have been caused by climatic differences between the two sampling years (Cocagne in 2021, Bouctouche in 2022). 2022 reached record high sea-surface temperatures (since time-series began in 1991) and warmed to summer temperatures two-weeks earlier than 2021 (74,75). Additionally, the month of August experienced 175 % (Government of Canada Historical Climate Data, Kouchibouguac station) more precipitation in 2022 (181.9 mm) compared to 2021 (66.2 mm), which may have enhanced nutrient availability from terrestrial runoff in 2022, contributing to a much higher peak in phytoplankton abundance at Bouctouche, which was dominated by picoPC-cyanobacteria (Fig 7), a freshwater indicator (34). The differences observed in bacteria and plankton communities between these two embayments may therefore be driven by interannual climatic differences rather than, or in addition to, aquaculture impacts (bivalve grazing pressure, nutrient alterations, benthic-pelagic coupling). This observation reinforces that inter-bay comparisons require a monitoring design that overcomes natural

seasonal stochasticity. This may be accomplished by capturing seasonality when monitoring inter-year changes (e.g., sampling at multiple periods) or limiting inter-year variation when contrasting ecosystems between embayments (e.g., sampling the same year at the same periods).

Organic resources and ecosystem functioning

Plankton abundance and relative abundance data are important for informing community structure and biodiversity, as well as having the ability to be correlated to changes in environmental conditions (39,40). However, they are not directly representative of the food and energy availability to higher trophic levels, as biomass and nutritional content varies between taxonomic groups. Information on plankton biomass, which is often estimated as a function of carbon content, is thus essential to better understand and model aquaculture ecosystem interactions and implications for higher trophic levels. The average carbon content of the cells analyzed using flow cytometry spans several orders of magnitude, with coastal heterotrophic bacteria estimated at 0.03 pg C $cell^{-1}$ (76) and cyanobacteria and picoeukaryotes estimated at 0.1 and 1.5 pg C $cell^{-1}$, respectively (77,78). Estimating carbon content of nanoeukaryotes is more challenging as it depends on biovolume (79,80), which must account for highly variable cell size and shape. However, with estimates exceeding 100 pg C $cell^{-1}$, nanoeukaryotes often dominate cell biomass < 20 µm, even when their relative abundance is low.

Biomass is therefore an important consideration to interpret plankton seasonality and timing of the summer phytoplankton bloom in embayments such as Bouctouche, which showed a strong seasonal pattern. Phytoplankton abundance was greatest in August due to high numbers of picoplankton, but phytoplankton biomass may have been greatest in July due to the increased contribution of nanoeukaryotes (> 73 % picophytoplankton in July, > 86 % picophytoplankton in

August). Phytoplankton biomass may also be estimated using bulk filtrations of POM and chl *a*, using filters of different porosity to quantify size classes (pico, nano, micro). However, the lack of relationship between chl *a* and POM concentrations, as well as chl *a* and phytoplankton abundance (Fig 13 D-E), indicate that organic pools at Bouctouche were not driven by phytoplankton < 20 µm, but likely had significant contributions from other marine and terrestrial plants (e.g., eelgrass beds), resuspended organic matter, fecal matter, or larger plankton. Microscopy counts or imaging technologies could additionally be used to quantify nano- and micro-phytoplankton and their biovolume for continued understanding of ecosystem functioning.

Nutrient effects

Picophytoplankton composed 40 to 80 % of total autotrophic biomass in enclosed shallow embayments (Bouctouche and Cocagne; Fig 11E). Using data compiled from 38 published reports spanning a wide range of environments, Agawin et al. (81) determined that picophytoplankton generally contribute > 50 % of autotrophic biomass in warm (> 26 °C), nutrient-poor (nitrate + nitrate < 1 µM) waters. Therefore the significant proportions of picophytoplankton observed here may be due to local nitrate and nitrite depletion (S1 Fig). Smaller phytoplankton cells perform better and tend to dominate under nutrient-limited conditions due to their higher surface area to volume ratio, which allows for more efficient nutrient acquisition (82,83). Previous studies conducted in aquaculture embayments have reported increased relative abundance (84) and biomass (2,3,85) of picophytoplankton due to increased ingestion efficiency of cells > 4 µm during bivalve filtration (12–15); however, this trend was not observed here. In fact, within-bay comparisons of stations showed no significant difference in picophytoplankton biomass between stations at Bouctouche, while at Cocagne, the North station had significantly lower picophytoplankton biomass, mainly driven by low biomass

recorded in June (Figs 11E and 12E). In conjunction, relative abundances of picophytoplankton, determined by flow cytometry, were lowest at the North and Inner stations within all embayments (Figs 6D and 10D). We therefore hypothesize that direct bivalve grazing, which would have increased picophytoplankton, is not the primary driver of picophytoplankton spatial patterns within the embayments studied here, but rather nutrient limitation, particularly nitrate and nitrite.

Notably, bivalve aquaculture sites may serve as either net sinks or sources of nitrogen depending on hydrodynamics, farming practices, sediment conditions, sediment resuspension rates and strength of benthic-pelagic coupling (86–88). Benthic-pelagic coupling may reintroduce remineralized nutrients from sediments into the pelagic ecosystem and stimulate primary production (89). Moving forward, results highlight the importance to assess the spatio-temporal variation of inorganic nutrients in conjunction with plankton to investigate ecosystem effects due to nutrient alterations within bivalve aquaculture embayments.

The contribution of HNA bacterial cells to total heterotrophic bacteria was additionally measured as a proxy for nutrient conditions, which showed the highest HNA proportions, observed in the shallow enclosed embayments, near-lease in late spring (May, June) and late summer (August, September), as well as near riverine inputs. These results showed high spatio-temporal variability, particularly as a function of seasonality, limiting the assessment of aquaculture effects and emphasizing the need for higher spatial resolution (e.g. under and near-lease) and replication. Whereas HNA determination from flow cytometry may be better used as a rapid and cost-efficient indicator method to observe potential long-term bay-scale changes in bacterial communities, molecular methods such as metabarcoding and quantitative PCR would

allow a more in depth characterization of bacteria composition and determination of functional genes and thus better inform biogeochemical cycling processes occurring near-lease (90–92).

Aquaculture interactions

Country Harbour is the only embayment in the study with no bivalve aquaculture grazing pressure due to empty leases at the time of sampling, and total phytoplankton cell abundance decreased from the Inner to Outer stations (Fig 6). This aligns with previous studies done in coastal bays, which have reported an inner to outer bay decrease in summer phytoplankton abundance along freshwater and nutrient gradients (93–95). In contrast to Country Harbour, the three other embayments, which contained several active leases, showed no significant difference in phytoplankton abundance along the bay gradient. We hypothesize that the relatively greater number of oyster leases at the Inner (Whitehead) and North (Bouctouche, Cocagne) stations may lead to increased consumption of phytoplankton cells, thereby reducing phytoplankton abundance and altering the natural inner to outer bay gradient. Additionally, hydrodynamic models have shown that the North stations at the shallow enclosed embayments have the highest water residence times (Bouctouche: 60.3 and 49.7 days at North and Central stations, respectively. Cocagne: 20.2, 14.4 and 19.2 days at North, Central and South stations, respectively; Guyondet, unpublished). This may decrease the rate at which additional phytoplankton cells are replenished and increase the vulnerability of these areas to aquaculture grazing pressure. Long-term monitoring through changes in aquaculture production within selected embayments may allow successful differentiation between spatially confounding factors from aquaculture (grazing, nutrient alteration and benthic-pelagic coupling) and natural coastal dynamics (water residence time, nutrient inputs and plankton uptake).

Within-bay patterns additionally showed that the stations most influenced by leases had the highest bacteria abundance, supporting that bacteria growth may be promoted by aquaculture activity due to increased organic nutrient availability through both dissolved and particulate excretion (96,97). Further studies are required to investigate if bacterial remineralization may enhance local inorganic nutrient availability for phytoplankton growth and auto-regulate primary production in relation to aquaculture level and local dynamics, and/or if long-term nutrient alteration and energy redirection towards the microbial loop may ultimately limit energy transfer to higher pelagic trophic levels (98–100).

Recommendations for monitoring

Here, we provide an overview of factors to be considered when monitoring bacteria and phytoplankton at the bay-scale in aquaculture embayments. Although the proportion of picophytoplankton has previously been proposed as an indicator of bivalve grazing, we observed that spatio-temporal trends in picophytoplankton were driven by nutrient gradients, limiting the ability to disentangle bivalve grazing pressure and aquaculture-related nutrient alterations from other spatial and seasonal variation. To avoid misinterpreting ecosystem changes as a result of aquaculture, seasonality must be considered with sampling seasons spanning at least 3 to 4 months to capture plankton dynamics and account for seasonal phenology shifts expected from climate change, and variability of grazing rates. To assess bay-scale composition, other local factors such as tide phase should be accounted for in areas where riverine vs marine influence may affect phytoplankton composition. Additionally, where the water column is stratified, water samples should be collected at multiple depths for a comprehensive understanding of plankton dynamics. To successfully detect long-term bay-scale changes in relation to aquaculture and

differentiate confounding factors, we recommend using a multi-metric approach to assess and forecast holistic bay-scale changes of ecosystem functioning. In addition to flow cytometry abundance data, plankton biomass is important for informing food and energy resources for higher trophic levels, and may be estimated using bulk filtrations of POM and chl *a*, using filters of different porosity to quantify size classes (pico, nano, micro). Finally, disentangling the effect of aquaculture pressure from stochastic processes, eutrophication, climate change, and fisheries would require appropriate reference sites. Assessing and forecasting aquaculture management thresholds require a better understanding of aquaculture production levels and will further require monitoring ecosystem changes over a gradient of aquaculture production in similar ecosystems (i.e., plankton composition, hydrodynamic regime) to reduce effects from confounding factors (101).

Conclusions

There is a need to better understand aquaculture ecosystem interactions and to develop predictive carrying capacity models to avoid a state where the role and functions of plankton would be substantially altered by cultured bivalve dominance. Here, empirical results show that bivalve grazing may reduce near-lease phytoplankton abundance and favor bacterial growth. However, it is unknown if ecosystem functioning is altered at the bay-scale. For example, it remains unclear if near-lease benthic-pelagic coupling may enhance bacterial remineralization of organic nutrients, made available through dissolved and particulate excretion from cultured bivalves, and auto-regulate primary production and/or limit energy transfer to higher trophic levels. Overall, environmental forcings appeared to be the main drivers of spatio-temporal trends in phytoplankton composition, which showed strong variation as a function of riverine vs

freshwater influence and nutrient limitations. Therefore, a long-term aquaculture monitoring program assessing planktonic communities must be carefully designed to be able to disentangle natural and anthropogenic factors to adequately support management decision-making.

Acknowledgements

Thanks to the Aquaculture Monitoring Program (AMP) working group members, the Advisory Committee, and program management for exchanging scientific ideas on this project, including Johannie Duhaime who coordinated AMP. Thank you to Lara Cooper for help with funding acquisition and program management. Thank you to Ramon Filgueira for his help with sampling design and logistics. Khang Hua for reviewing sampling protocol and compiling and providing the data and metadata. Thank you to Stephen Finnis for metadata compilation and guidance with statistical analysis and R scripts. We thank Brian Fortune for his support to access the Country Harbour and Whitehead sites, logistics, and aquaculture information. Thank you to Julie Arseneau and Steven Neil for their help with sample collection at Country Harbour and Whitehead, and to Jeffery Clements, Johannie Duhaime, Michael Coffin, Katherine Gingles, Sarah Harrison, Julie LeRay, Rémi Sonier, and Véronic St-Laurent for assistance with fieldwork at Bouctouche and Cocagne.

References

1. Norén F, Haamer J, Lindahl O. Changes in the plankton community passing a *Mytilus edulis* bed. Mar Ecol Prog Ser. 1999;191:187–94.

2. Souchu P, Vaquer A, Collos Y, Landrein S, Deslous-Paoli J, Bibent B. Influence of shellfish farming activities on the biogeochemical composition of the water column in Thau lagoon. Mar Ecol Prog Ser. 2001;218:141–52.

3. Jiang T, Chen F, Yu Z, Lu L, Wang Z. Size-dependent depletion and community disturbance of phytoplankton under intensive oyster mariculture based on HPLC pigment analysis in Daya Bay, South China Sea. Environ Pollut. 2016;219:804–14.

4. Trottet A, Roy S, Tamigneaux E, Lovejoy C, Tremblay R. Impact of suspended mussels (*Mytilus edulis* L.) on plankton communities in a Magdalen Islands lagoon (Québec, Canada): A mesocosm approach. J Exp Mar Biol Ecol. 2008;365(2):103–15.

5. Sonier R, Filgueira R, Guyondet T, Tremblay R, Olivier F, Meziane T, et al. Picophytoplankton contribution to *Mytilus edulis* growth in an intensive culture environment. Mar Biol. 2016;163(4):73.

6. Shumway SE, Cucci TL, Newell RC, Yentsch CM. Particle selection, ingestion, and absorption in filter-feeding bivalves. J Exp Mar Biol Ecol. 1985;91(1–2):77–92.

7. Ward JE, Shumway SE. Separating the grain from the chaff: particle selection in suspension- and deposit-feeding bivalves. J Exp Mar Biol Ecol. 2004;300(1–2):83–130.

8. Beninger PG, Valdizan A, Decottignies P, Cognie B. Impact of seston characteristics on qualitative particle selection sites and efficiencies in the pseudolamellibranch bivalve *Crassostrea gigas*. J Exp Mar Biol Ecol. 2008;360(1):9–14.

9. Strohmeier T, Strand Ø, Alunno-Bruscia M, Duinker A, Cranford PJ. Variability in particle retention efficiency by the mussel *Mytilus edulis*. J Exp Mar Biol Ecol. 2012;412:96–102.

10. Rosa M, Ward JE, Shumway SE. Examining the effects of microalgal metabolites on ciliary activity of the Eastern Oyster *Crassostrea virginica*. J Shellfish Res. 2021;40(2):231–7.

11. Rosa M, Ward JE, Shumway SE. Selective capture and ingestion of particles by suspension-feeding bivalve molluscs: A review. J Shellfish Res. 2018;37(4):727–46.

12. Morton B. Feeding and digestion in bivalvia. In: Saleuddin A, Wilbur K, editors. The mollusca. New York: Academic Press; 1983. p. 65–147.

13. McHenery JG, Birkbeck TH. Uptake and processing of cultured microorganisms by bivalves. J Exp Mar Biol Ecol. 1985;90(2):145–63.

14. Riisgård H. Efficiency of particle retention and filtration rate in 6 species of Northeast American bivalves. Mar Ecol Prog Ser. 1988;45:217–23.

15. Riisgård H, Larsen P. Particle capture mechanisms in suspension-feeding invertebrates. Mar Ecol Prog Ser. 2010;418:255–93.

16. Nevejan N, De Schryver P, Wille M, Dierckens K, Baruah K, Van Stappen G. Bacteria as food in aquaculture: do they make a difference? Rev Aquac. 2018;10(1):180–212.

17. Walne P. Studies on the food value of nineteen genera of algae to juvenile bivalves of the genera *Ostrea, Crassostrea, Mercenaria* and *Mytilus*. Fish Invest Lond Ser. 1970;26:1–62.

18. Webb KL, Chu FE. Phytoplankton as a food source for bivalve larve. In: Biogeochemical and physical approaches to shellfish nutrition. Baton Rouge: Louisiana State University Press; 1983. p. 272–91.

19. Enright CT, Newkirk GF, Craigie JS, Castell JD. Evaluation of phytoplankton as diets for juvenile *Ostrea edulis* L. J Exp Mar Biol Ecol. 1986;96(1):1–13.

20. Gosling E. Bivalve molluscs: Biology, ecology and culture. Oxford: Blackwell Publishing Ltd; 2003.

21. Langdon CJ, Newell RIE. Utilization of detritus and bacteria as food sources by two bivalve suspension-feeders, the oyster *Crassostrea virginica* and the mussel *Geukensia demissa*. Mar Ecol Prog Ser. 1989;58:299–310.

22. Wangersky PJ. The role of particulate matter in the productivity of surface waters. Helgol Wiss Meeresunters. 1977;30:546–64.

23. Sorokin YI. Microheterotrophic organisms in marine ecosystems. In: Longhurts AR, editor. Analysis of marine ecosystems. London: Academic Press; 1981. p. 293–342.

24. Wotton RS. The ubiquity and many roles of exopolymers (EPS) in aquatic systems. Sci Mar. 2004;68:13–21.

25. McKee MP, Ward JE, MacDonald BA, Holohan BA. Production of transparent exopolymer particles (TEP) by the eastern oyster (*Crassostrea virginica*). Mar Ecol Prog Ser. 2005;288:141–9.

26. Heinonen KB, Ward JE, Holohan BA. Production of transparent exopolymer particles (TEP) by benthic suspension feeders in coastal systems. J Exp Mar Biol Ecol. 2007;341(2):184–95.

27. Stuart V, Newell RC, Lucas MI. Conversion of kelp debris and faecal material from the mussel *Aulacomya ater* by marine micro-organisms. Mar Ecol Prog Ser. 1982;7:47–57.

28. Seiderer LJ, Newell RC. Relative significance of phytoplankton, bacteria and plankton detritus as carbon and nitrogen resources for the kelp bed filter-feeder *Chromytilus meridionalis*. Mar Ecol Prog Ser. 1985;22:127–39.

29. Legendre L, Courties C, Troussellier M. Flow cytometry in oceanography 1989-1999: Environmental challenges and research trends. Cytometry. 2001;44(3):164–72.

30. Van Nevel S, Koetzsch S, Proctor CR, Besmer MD, Prest EI, Vrouwenvelder JS, et al. Flow cytometric bacterial cell counts challenge conventional heterotrophic plate counts for routine microbiological drinking water monitoring. Water Res. 2017;113:191–206.

31. Yentsch CM, Horan PK, Muirhead K, Dortch Q, Haugen E, Legendre L, et al. Flow cytometry and cell sorting: A technique for analysis and sorting of aquatic particles. Limnol Oceanogr. 1983;28(6):1275–80.

32. Rutten TPA, Sandee B, Hofman ART. Phytoplankton monitoring by high performance flow cytometry: A successful approach? Cytometry A. 2005;64A(1):16–26.

33. Malkassian A, Nerini D, Van Dijk MA, Thyssen M, Mante C, Gregori G. Functional analysis and classification of phytoplankton based on data from an automated flow cytometer. Cytometry A. 2011;79A(4):263–75.

34. Poniedziałek B, Falfushynska HI, Rzymski P. Flow cytometry as a valuable tool to study cyanobacteria:A mini-review. Limnol Rev. 2017;17(2):89–95.

35. Jacobs P, Riegman R, van der Meer J. Impact of introduced juvenile mussel cultures on the pelagic ecosystem of the western Wadden Sea, The Netherlands. Aquac Environ Interact. 2016;8:553–6.

36. Fournier J, Dupuy C, Bouvy M, Couraudon-Réale M, Charpy L, Pouvreau S, et al. Pearl oysters *Pinctada margaritifera* grazing on natural plankton in Ahe atoll lagoon (Tuamotu archipelago, French Polynesia. Mar Pollut Bull. 2012;65:490–9.

37. Bougrier S, Hawkins AJS, Heral M. Preingestive selection of different microalgal mixtures in *Crassostrea gigas* and *Mytilus edulis,* analysed by flow cytometry. Aquaculture. 1997;150:123–34.

38. Dupuis C, Vaquer A, Lam-Höai T, Rougier C, Mazouni N, Lautier J, et al. Feeding rate of the oyster *Crassostrea gigas* in a natural planktonic community of the Mediterranean Thau Lagoon. Mar Ecol Prog Ser. 2000;205:171–84.

39. Laux M, Torgan LC. Which metric to choose? Differences between abundance and biomass responses to environmental conditions in a planktonic diatom community. Hydrobiologia. 2015;744(1):63–76.

40. Ní Longphuirt S, McDermott G, O’Boyle S, Wilkes R, Stengel DB. Decoupling abundance and biomass of phytoplankton communities under different environmental controls: A new multi-metric index. Front Mar Sci. 2019;6:312.

41. Gasol JM, Zweifel UL, Peters F, Fuhrman JA, Hagström Å. Significance of size and nucleic acid content heterogeneity as measured by flow cytometry in natural planktonic bacteria. Appl Environ Microbiol. 1999;65(10):4475–83.

42. Bouvier T, Del Giorgio PA, Gasol JM. A comparative study of the cytometric characteristics of high and low nucleic-acid bacterioplankton cells from different aquatic ecosystems. Environ Microbiol. 2007;9(8):2050–66.

43. De Roy K, Clement L, Thas O, Wang Y, Boon N. Flow cytometry for fast microbial community fingerprinting. Water Res. 2012;46(3):907–19.

44. Romdhane SB, Bour ME, Hamza A, Akrout F, Kraiem MM, Jacquet S. Seasonal patterns of viral, microbial and planktonic communities in Sidi Salem: a freshwater reservoir (North of Tunisia). Ann Limnol - Int J Lim. 2014;50(4):299–314.

45. Santos M, Oliveira H, Pereira JL, Pereira MJ, Gonçalves FJM, Vidal T. Flow cytometry analysis of low/high DNA content (LNA/HNA) bacteria as bioindicator of water quality evaluation. Ecol Indic. 2019;103:774–81.

46. Giovannoni SJ, Tripp HJ, Givan S, Podar M, Vergin KL, Baptista D, et al. Genome streamlining in a cosmopolitan oceanic bacterium. Science. 2005;309:1242–5.

47. Giovannoni SJ. SAR11 Bacteria: The most abundance plankton in the oceans. Annu Rev Mar Sci. 2017;3(9):231–55.

48. Servais P, Casamayor EO, Courties C, Catala P, Parthuisot N, Lebaron P. Activity and diversity of bacterial cells with high and low nucleic acid content. Aquat Microb Ecol. 2003;33:41–51.

49. Longnecker K, Sherr BF, Sherr EB. Activity and phylogenetic diversity of bacterial cells with high and low nucleic acid content and electron transport system activity in an upwelling ecosystem. Appl Environ Microbiol. 2005;71(12):7737–49.

50. Longnecker K, Sherr BF, Sherr EB. Variation in cell-specific rates of leucine and thymidine incorporation by marine bacteria with high and with low nucleic acid content off the Oregon coast. Aquat Microb Ecol. 2006;43:113–25.

51. Mojica KDA, Carlson CA, Behrenfeld MJ. Regulation of low and high nucleic acid fluorescent heterotrophic prokaryote subpopulations and links to viral-induced mortality within natural prokaryote-virus communities. Microb Ecol. 2020;79(1):213–30.

52. Hu C, Chen X, Yu L, Xu D, Jiao N. Elevated contribution of low nucleic acid prokaryotes and viral lysis to the prokaryotic community along the nutrient gradient from an estuary to open ocean transect. Front Microbiol. 2020;11:612053.

53. Shapiro HM. Practical Flow Cytometry. 4th ed. New York: Wiley-Liss; 2003.

54. Mella-Flores D, Six C, Ratin M, Partensky F, Boutte C, Le Corguillé G, et al. *Prochlorococcus* and *Synechococcus* have evolved different adaptive mechanisms to cope with light and UV stress. Front Microbiol. 2012;3.

55. Gianasi B, McKindsey C, Tremblay R, Comeau L, Drolet D. Plankton depletion by mussel grazing negatively impacts the fitness of lobster larvae. Aquaculture. 2023;574:739659.

56. Finnis S, Guyondet T, McKindsey CW, Arseneau J, Barrell J, Duhaime J, et al. Guidance on sampling effort to monitor mesozooplankton communities at Canadian bivalve aquaculture sites using an optical imaging system. Fisheries and Oceans Canada; 2023 p. vii + 101. (Canadian Technical Report of Fisheries and Aquatic Science). Report No.: 3581.

57. Belzile C, Brugel S, Nozais C, Gratton Y, Demers S. Variations of the abundance and nucleic acid content of heterotrophic bacteria in Beaufort Shelf waters during winter and spring. J Mar Syst. 2008;74(3–4):946–56.

58. Araújo CAS, Belzile C, Tremblay JÉ, Bélanger S. Environmental niches and seasonal succession of phytoplankton assemblages in a subarctic coastal bay: Applications to remote sensing estimates. Front Mar Sci. 2022;9:1001098.

59. Kirk J. Light and photosynthesis in aquatic ecosystems. 2nd ed. Cambridge: Cambridge University Press; 1994. 509 p.

60. Arar EJ, Collins GB. In Vitro Determination of Chlorophyll a and Pheophytin a in Marine and Freshwater Algae by Fluorescence. National Exposure Research Laboratory Office of Research and Development U.S. Environmental Protection Agency Cincinnati, Ohio 45268; 1997.

61. Borg I, Groenen P. Modern multidimensional scaling: Theory and applications. 2nd ed. New York: Springer Science+ Business Media; 2005.

62. Oksanen J, Blanchet FG, Kindt R, Legendre P, Minchin PR, O'Hara R, et al. vegan: Community Ecology Package [Internet]. 2012. Available from: http://CRAN.R-project.org/package=vegan

63. Azam F, Fenchel T, Field JG, Gray JS, Meyer-Reil LA, Thingstad F. The ecological role of water-column microbes in the sea. Mar Ecol Prog Ser. 1983;10:257–63.

64. Legendre L, Rassoulzadegan F. Plankton and nutrient dynamics in marine waters. Ophelia. 1995;41(1):153–72.

65. Degerman R, Lefébure R, Byström P, Båmstedt U, Larsson S, Andersson A. Food web interactions determine energy transfer efficiency and top consumer responses to inputs of dissolved organic carbon. Hydrobiologia. 2018;805(1):131–46.

66. Guijarro-Sabaniel J, Kelly NE. Land use atlas for coastal watersheds in the Maritimes Region. Fisheries and Oceans Canada; 2022 p. viii + 135. (Canadian Technical Report of Fisheries and Aquatic Science). Report No.: 3493.

67. Sharpe H, Gallardi D, Gurney-Smith H, Guyondet T, McKindsey CW, Lacoursière-Roussel A. Ecological insight of seasonal plankton succession to monitor shellfish aquaculture ecosystem interactions. Front Mar Sci. 2024;

68. Therriault JC, Levasseur M. Control of phytoplankton production in the Lower St. Lawrence Estuary: light and freshwater runoff. Nat Can. 1985;112:77–96.

69. Matsumoto K, Honda MC, Sasaoka K, Wakita M, Kawakami H, Watanabe S. Seasonal variability of primary production and phytoplankton biomass in the western Pacific subarctic gyre: Control by light availability within the mixed layer. J Geophys Res Oceans. 2014;119(9):6523–34.

70. Aota Y, Nakajima H. Mutualistic relationships between phytoplankton and bacteria caused by carbon excretion from phytoplankton. Ecol Res. 2001;16(2):289–99.

71. McManus GB, Peterson WT. Bacterioplankton production in the nearshore zone during upwelling off central Chile. Mar Ecol Prog Ser. 1988;43:11–7.

72. Bouvy M, Arfi R, Guiral D, Pagano M, Saint-Jean L. Role of bacteria as food for zooplankton in a eutrophic tropical pond (Ivory Coast). Netherland J Aquat Ecol. 1994;28(2):167–74.

73. Kim HW, Hwang SJ, Joo GJ. Zooplankton grazing on bacteria and phytoplankton in a regulated large river (Nakdong River, Korea). J Plankton Res. 2000;22:1559–77.

74. Galbraith PS, Chassé J, Dumas J, Shaw JL, Caverhill C, Lefaivre D, et al. Physical oceanographic conditions in the Gulf of St. Lawrence during 2021. Quebec Region: DFO; 2022 p. iv + 83. Report No.: 2022/034.

75. Galbraith PS, Chassé J, Shaw J-L, Dumas J, Lefaivre D, Bourassa M-N. Physical oceanographic conditions in the Gulf of St. Lawrence during 2022. Ottawa: DFO; 2023 p. v + 88. Report No.: 354.

76. Fukuda R, Ogawa H, Nagata T, Koike I. Direct determination of carbon and nitrogen contents of natural bacterial assemblages in marine environments. Appl Environ Microbiol. 1998;64(9):3352–8.

77. Zubkov MV, Sleigh MA, Tarran GA, Burkill PH, Leakey RJG. Picoplanktonic community structure on an Atlantic transect from 50°N to 50°S. Deep Sea Res Part Oceanogr Res Pap. 1998;45(8):1339–55.

78. Zubkov MV, Sleigh MA, Burkill PH, Leakey RJG. Picoplankton community structure on the Atlantic Meridional Transect: a comparison between seasons. Prog Oceanogr. 2000;45(3):369–86.

79. Garrison DL, Gowing MM, Hughes MP, Campbell L, Caron DA, Dennett MR, et al. Microbial food web structure in the Arabian Sea: a US JGOFS study. Deep Sea Res Part II Top Stud Oceanogr. 2000;47(7–8):1387–422.

80. Owen K. Flow cytometric investigation of the size spectrum of North Sea phytoplankton communities [PhD]. University of East Anglia; 2014.

81. Agawin NSR, Duarte CM, Agustí S. Nutrient and temperature control of the contribution of picoplankton to phytoplankton biomass and production. Limnol Oceanogr. 2000;45(3):591–600.

82. Malone T. Algal size. In: Morris I, editor. The physiological ecology of phytoplankton. Berkley: University of California Press; 1980. p. 433–63.

83. Chisholm SW. Phytoplankton size. In: Falkowski PG, Woodhead AD, Vivirito K, editors. Primary productivity and biogeochemical cycles in the sea. Boston, MA: Springer US; 1992. p. 213–37.

84. Vaquer A, Troussellier M, Courties C, Bibent B. Standing stock and dynamics of picophytoplankton in the Thau Lagoon (northwest Mediterranean coast). Limnol Oceanogr. 1996;41(8):1821–8.

85. Cranford PJ, Li W, Strand Ø, Strohmeier T. Phytoplankton depletion by mussel aquaculture: high resolution mapping, ecosystem modeling, and potential indicators of ecological carrying capacity. Int Counc Explor Seas. 2008; p. 5.

86. Filgueira R, Comeau LA, Guyondet T, McKindsey CW, Byron C. Modelling carrying capacity of bivalve aquaculture: A review of definitions and methods. In: Meyers R, editor. Encyclopedia of Sustainability Science and Technology. New York: Springer New York; 2015. p. 1–33.

87. Lacoste É, McKindsey CW, Archambault P. Biodiversity–Ecosystem Functioning (BEF) approach to further understanding aquaculture–environment interactions with application to bivalve culture and benthic ecosystems. Rev Aquac. 2020;12(4):2027–41.

88. Azra MN, Okomoda VT, Tabatabaei M, Hassan M, Ikhwanuddin M. The contributions of shellfish aquaculture to global food security: Assessing its characteristics from a future food perspective. Front Mar Sci. 2021;8:654897.

89. Nunnally CC. Benthic–pelagic coupling: Linkages between benthic ecology and biogeochemistry and pelagic ecosystems and process. In: Cochran JK, Bokuniewicz HJ, Yager PL, editors. Encyclopedia of ocean sciences 3rd ed. Oxford: Academic Press; 2019. p. 660–2.

90. Kondo R, Mori Y, Sakami T. Comparison of sulphate-reducing bacterial communities in Japanese fish farm sediments with different levels of organic enrichment. Microbes Environ. 2012;27(2):193–9.

91. Zhang Y, Wang X, Zhen Y, Mi T, He H, Yu Z. Microbial diversity and community structure of sulfate-reducing and sulfur-oxidizing bacteria in sediment cores from the East China Sea. Front Microbiol. 2017;8.

92. Cordier T, Alonso-Sáez L, Apothéloz-Perret-Gentil L, Aylagas E, Bohan DA, Bouchez A, et al. Ecosystems monitoring powered by environmental genomics: A review of current strategies with an implementation roadmap. Mol Ecol. 2021;30(13):2937–58.

93. Yuan M, Zhang C, Jiang Z, Guo S, Sun J. Seasonal variations in phytoplankton community structure in the Sanggou, Ailian, and Lidao Bays. J Ocean Univ China. 2014;13(6):1012–24.

94. Oseji OF, Chigbu P, Oghenekaro E, Waguespack Y, Chen N. Spatiotemporal patterns of phytoplankton composition and abundance in the Maryland Coastal Bays: The influence of freshwater discharge and anthropogenic activities. Estuar Coast Shelf Sci. 2018;207:119–31.

95. Gao Y, Jiang Z, Chen Y, Liu J, Zhu Y, Liu X, et al. Spatial variability of phytoplankton and environmental drivers in the turbid Sanmen Bay (East China Sea). Estuaries Coasts. 2022;45(8):2519–33.

96. Grant J, Hatcher A, Scott DB, Pocklington P, Schafer CT, Winters GV. A multidisciplinary approach to evaluating impacts of shellfish aquaculture on benthic communities. Estuaries. 1995;18(1):124–44.

97. Dame RF. Ecosystem of marine bivalves: An ecosystem approach. 1st ed. Boca Raton: CRC Press; 1996. 272 p.

98. Rodhouse PG, Roden CM. Carbon budget for a coastal inlet in relation to intensive cultivation of suspension-feeding bivalve molluscs. Mar Ecol Prog Ser. 1987;36:225–36.

99. Horsted SJ, Nielsen TG, Riemann B, Pock-Steen J, Bjørnsen PK. Regulation of zooplankton by suspension-feeding bivalves and fish in estuarine enclosures. Mar Ecol Prog Ser. 1988;48:217–24.

100. Hulot V, Saulnier D, Lafabrie C, Gaertner-Mazouni N. Shellfish culture: a complex driver of planktonic communities. Rev Aquac. 2020;12(1):33–46.

101. Cao Y, Wang L. How to statistically disentangle the effects of environmental factors and human disturbances: A review. Water. 2023;15(4).

Supporting information

S1 Fig. Inorganic nutrients concentrations from Bouctouche. (A) Phosphate, (B) nitrite and nitrate, (C) nitrate and (D) silicate. Water samples for inorganic nutrient concentrations were collected at the same time and locations as water samples for flow cytometry, however stations are unknown here.

Fig1

Click here to access/download;Figure;Fig1.tif

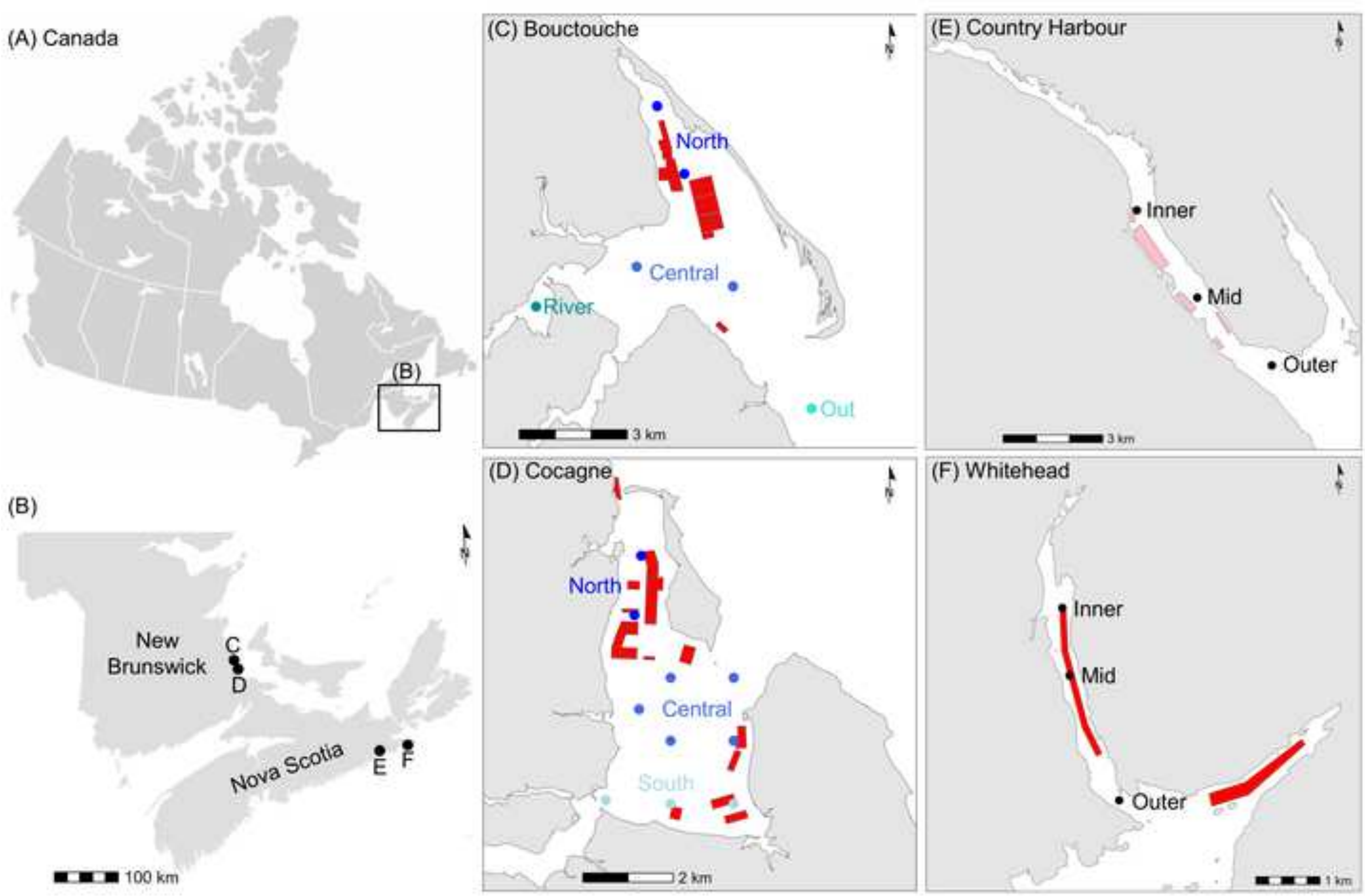

Fig2

Click here to access/download;Figure;Fig2.tif

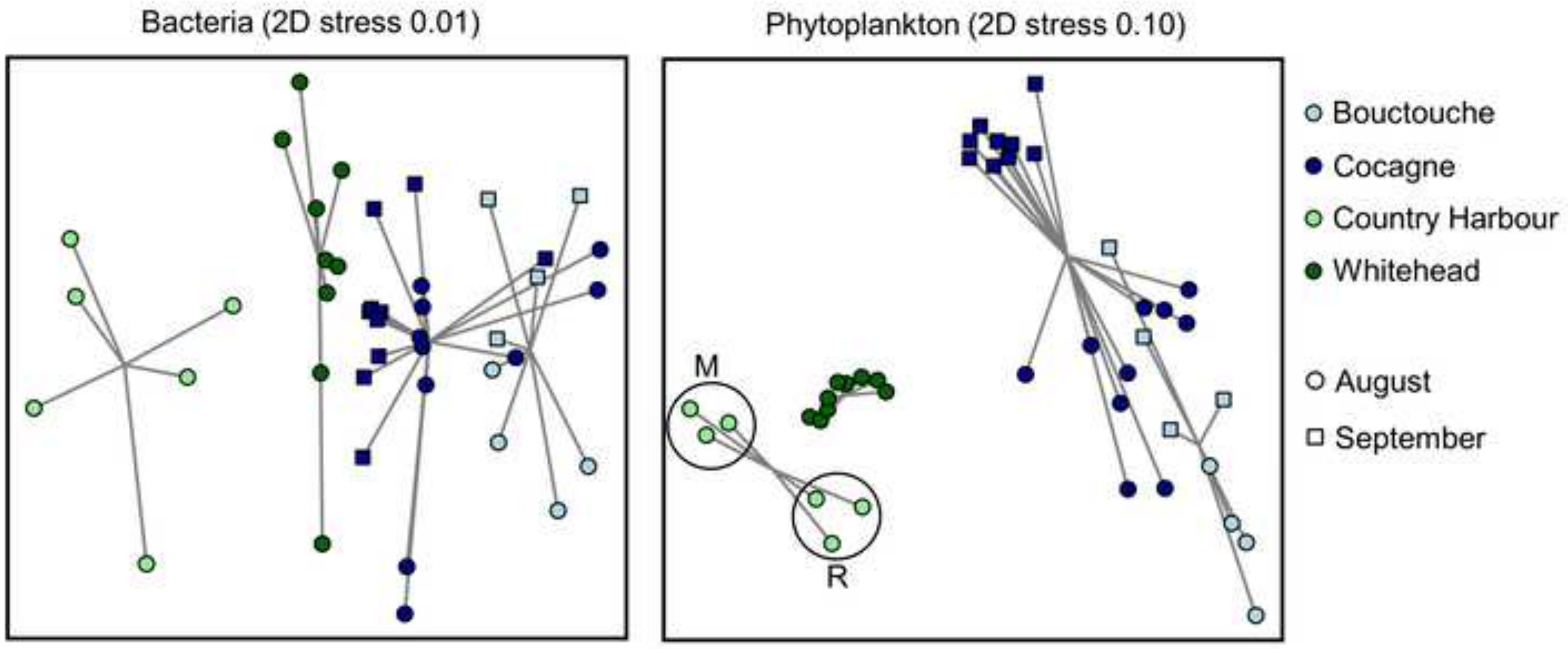

Fig3

Click here to access/download;Figure;Fig3.tif

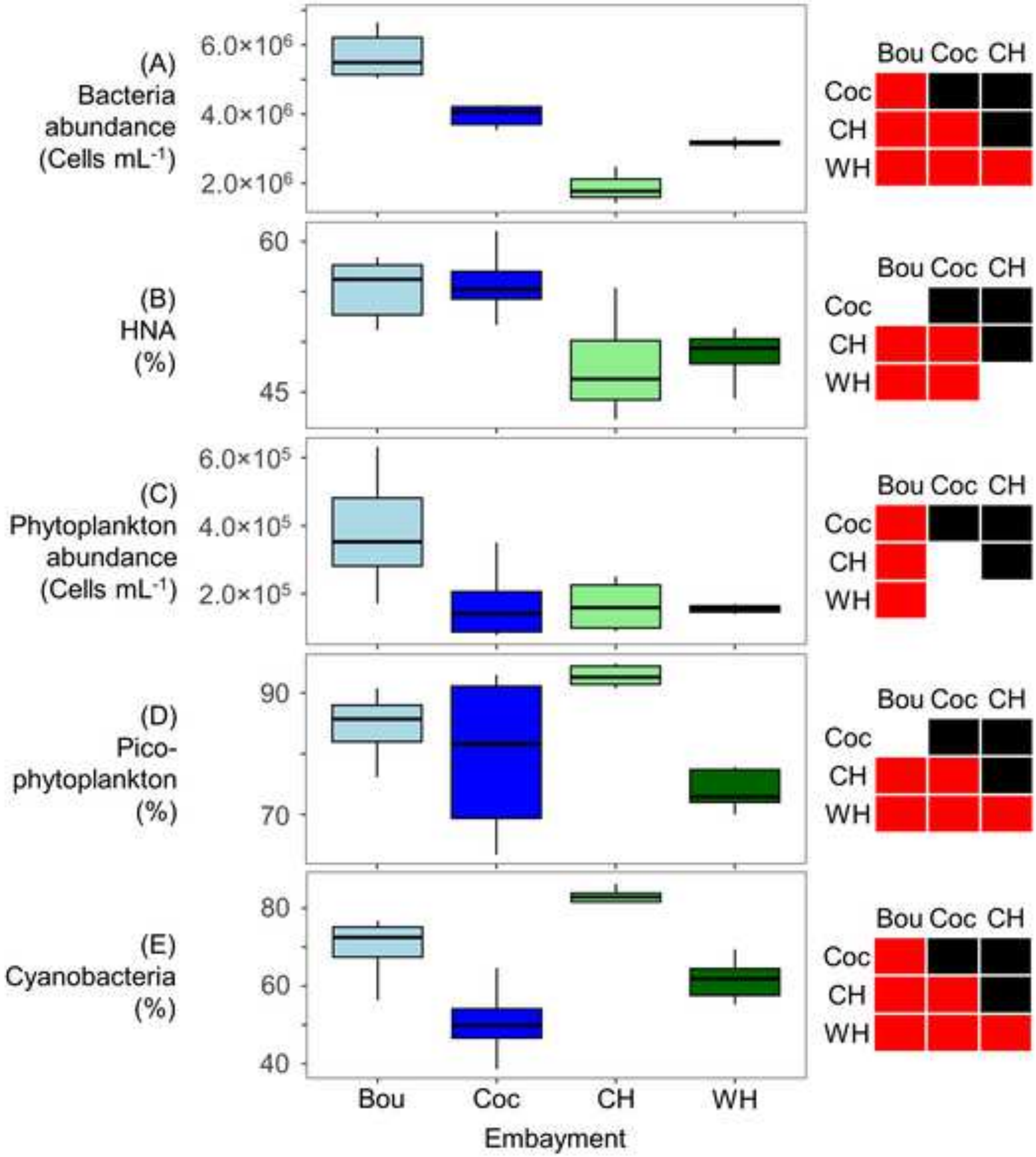

Fig4 Click here to access/download;Figure;Fig4.tif

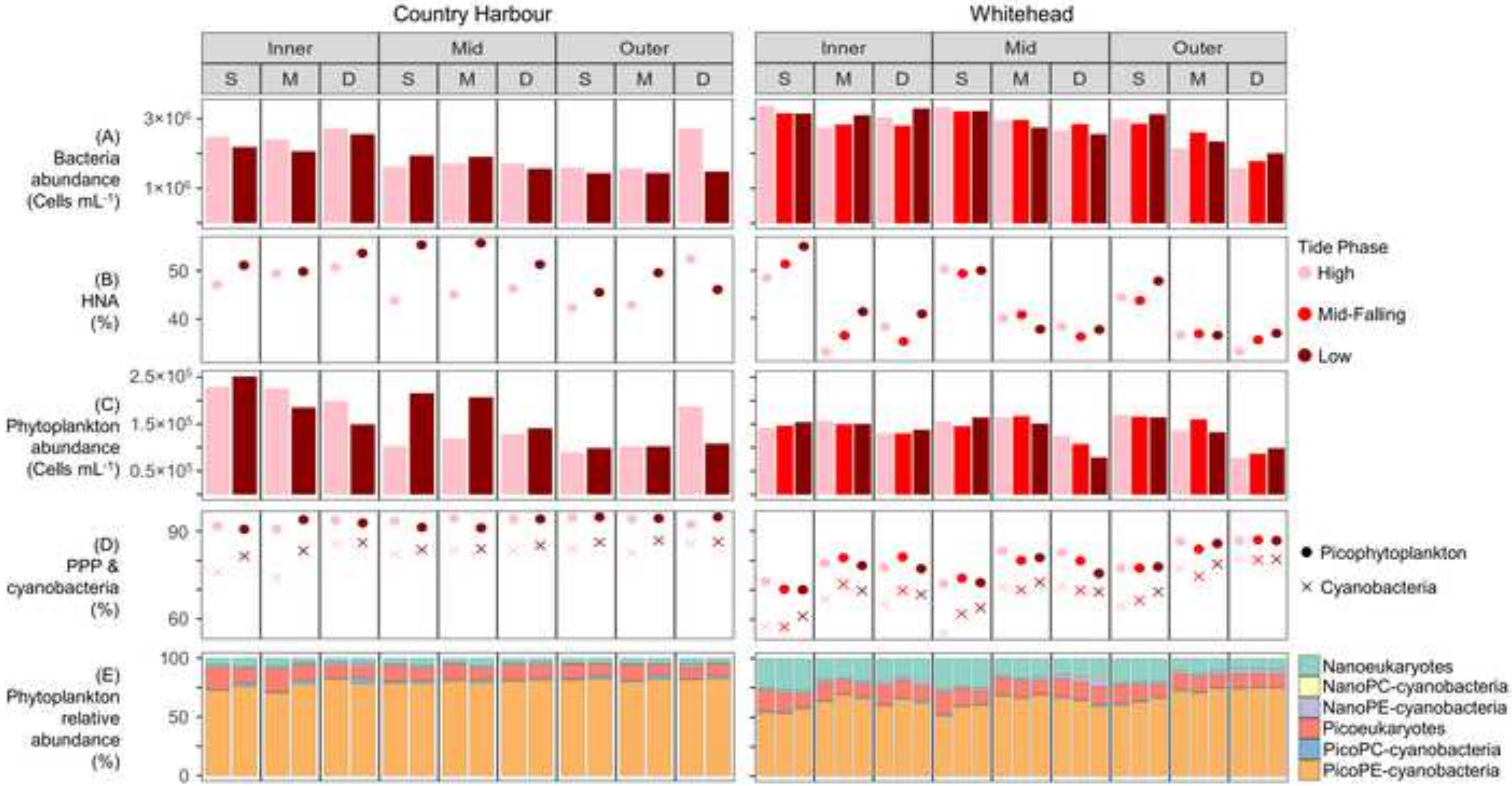

Fig5

Click here to access/download;Figure;Fig5.tif

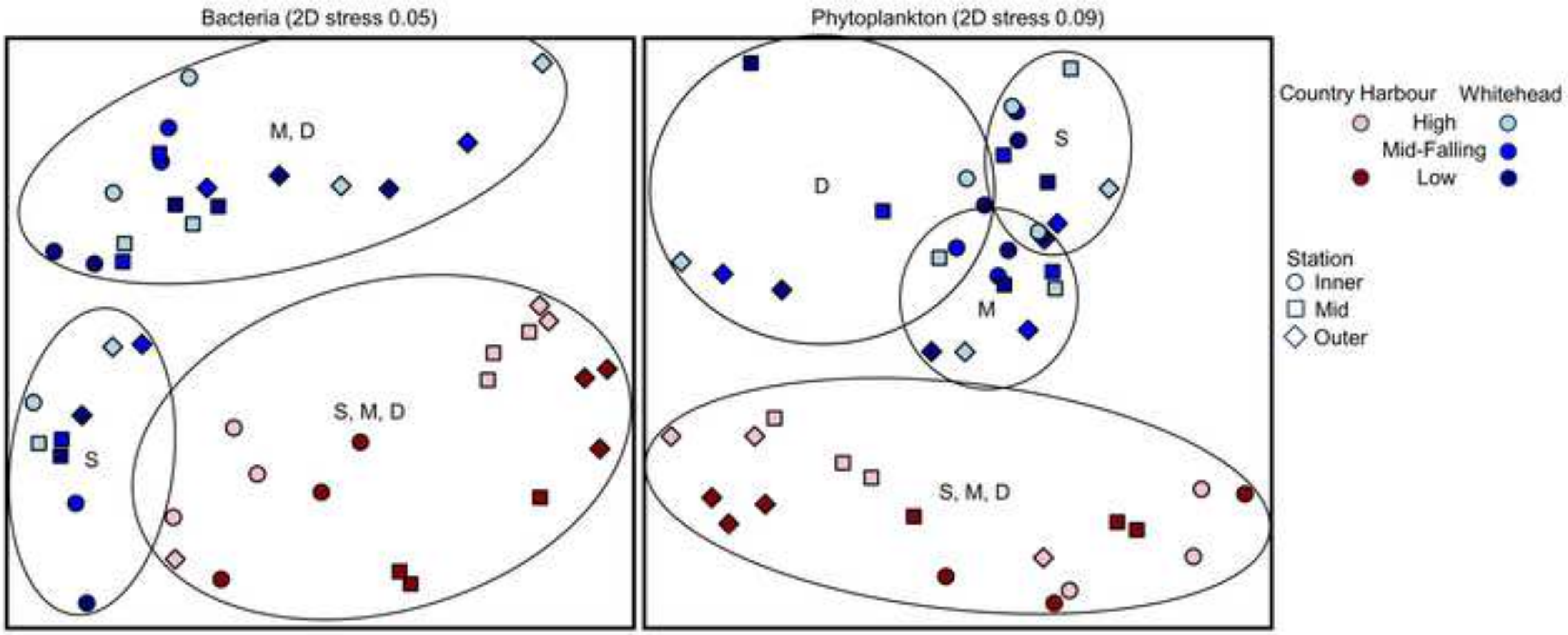

Fig6

Click here to access/download;Figure;Fig6.tif

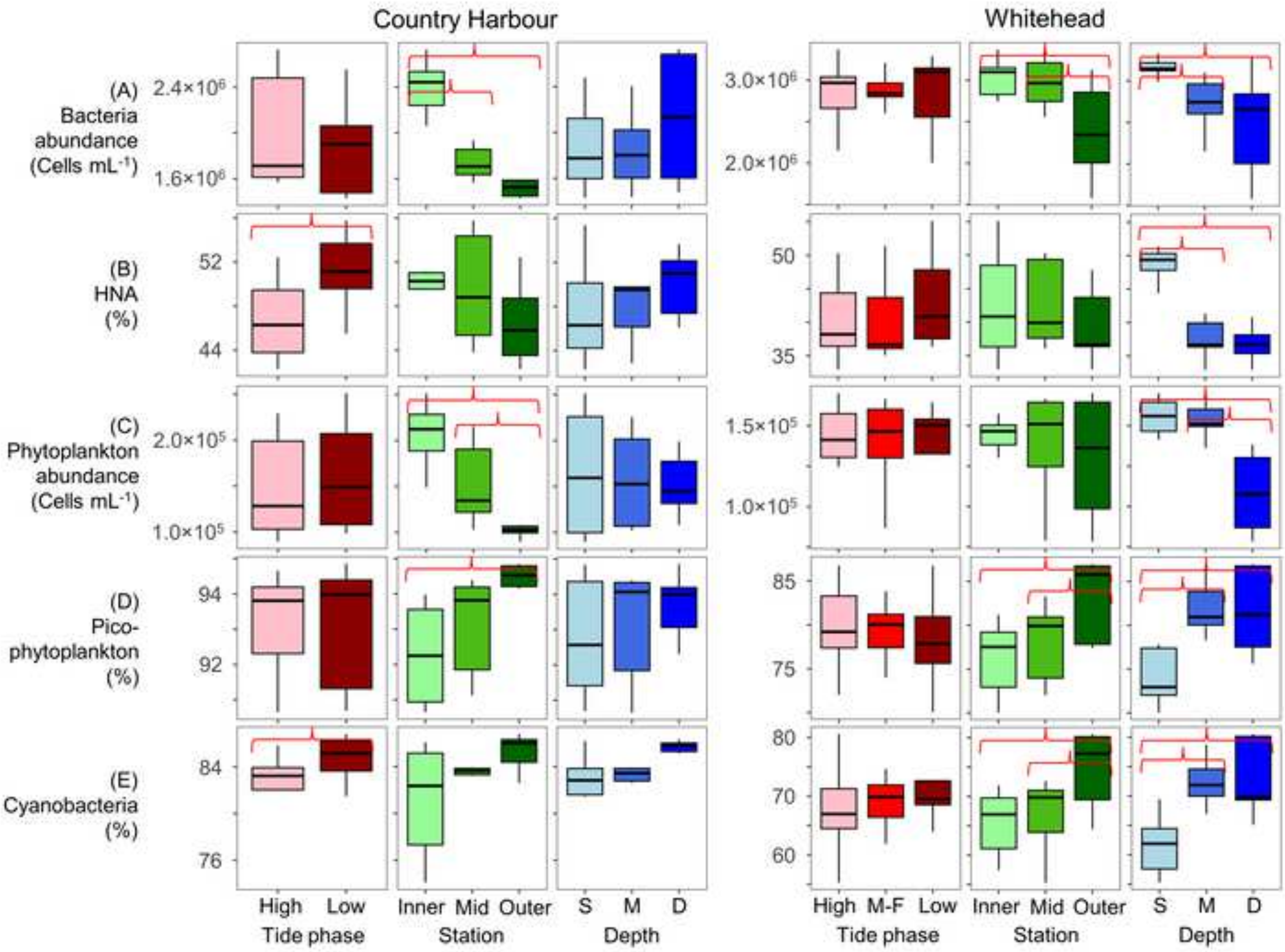

Fig7

Click here to access/download;Figure;Fig7.tif

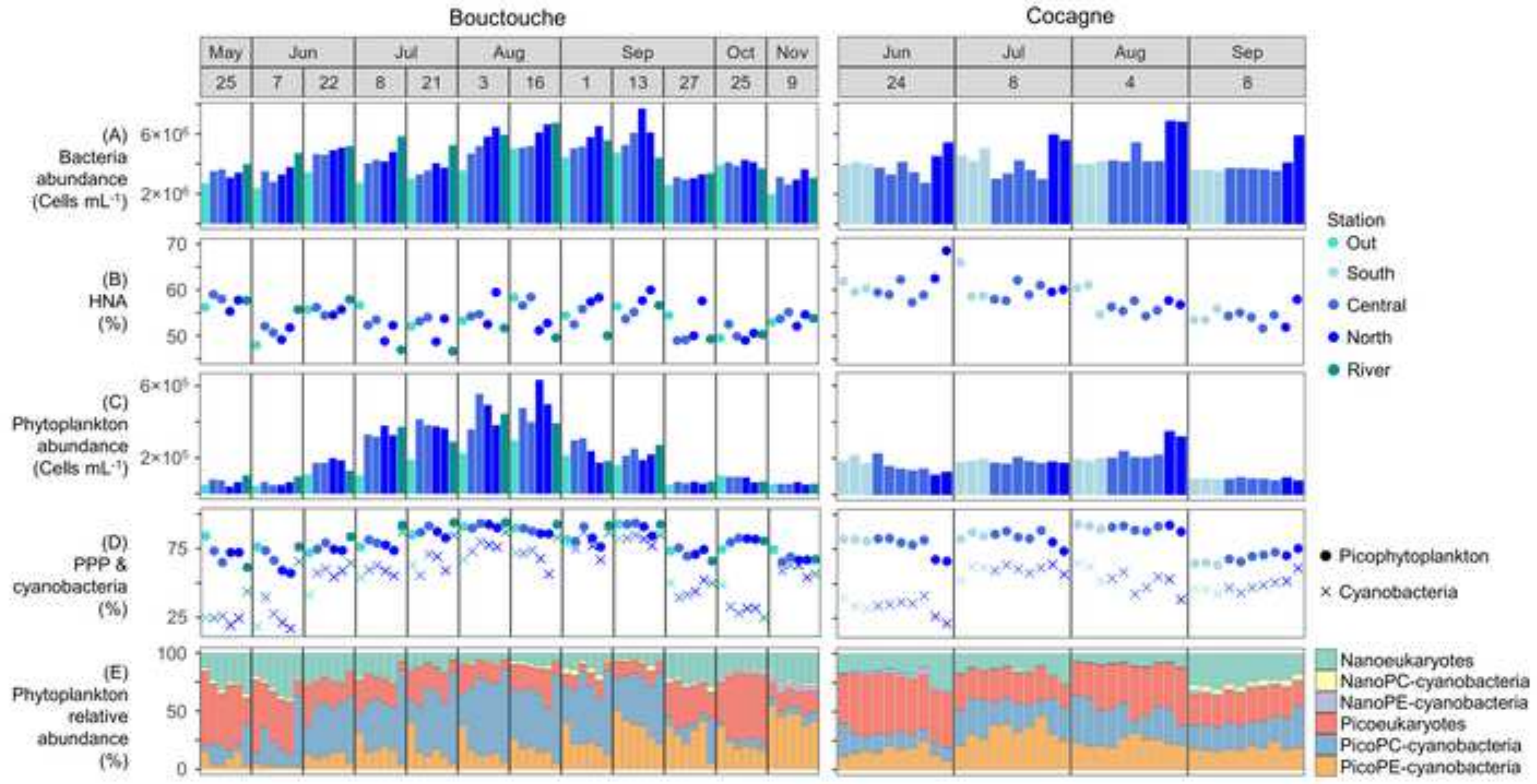

Click here to access/download;Figure;Fig8.tif

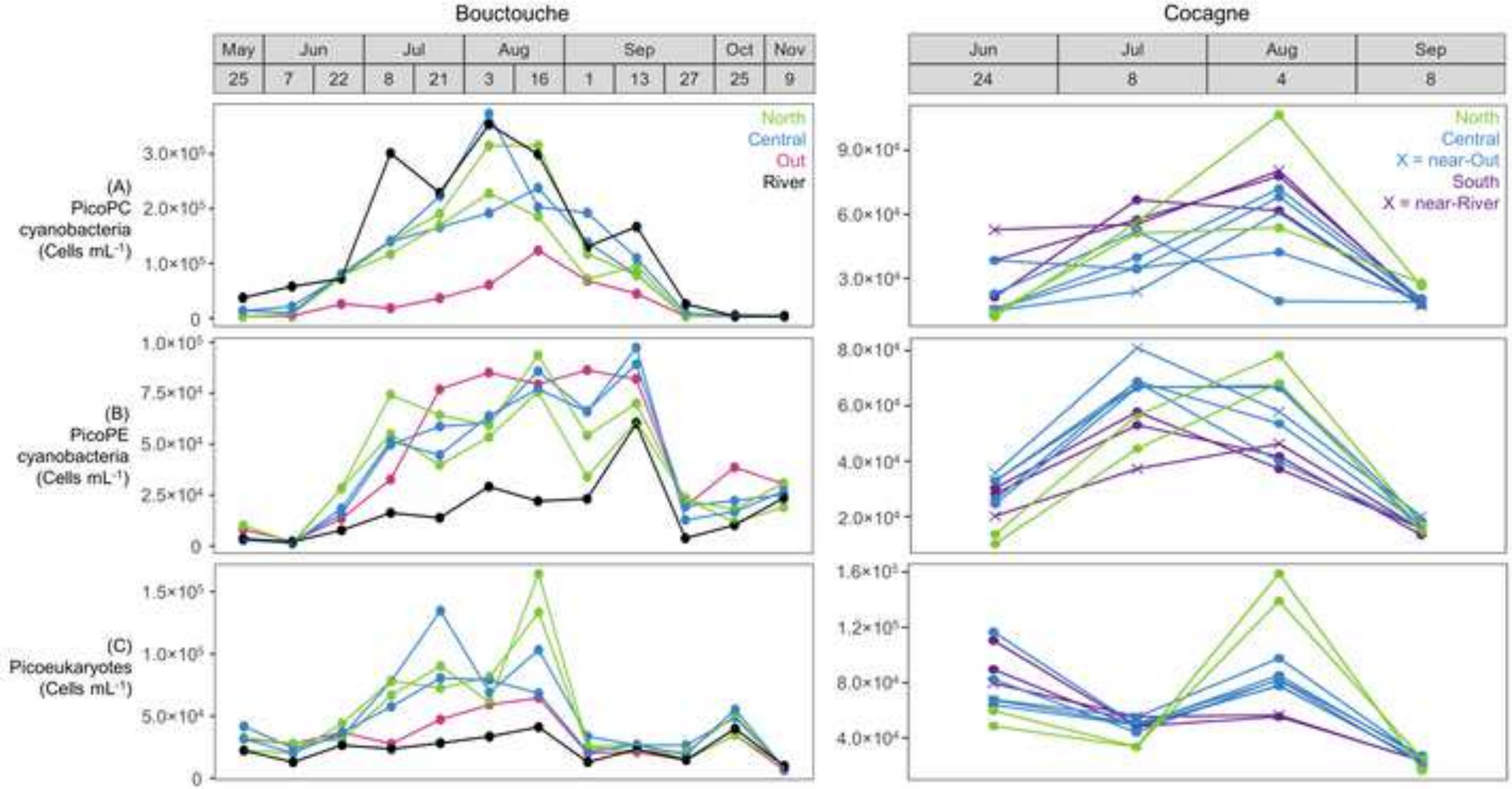

Fig9

Click here to access/download;Figure;Fig9.tif

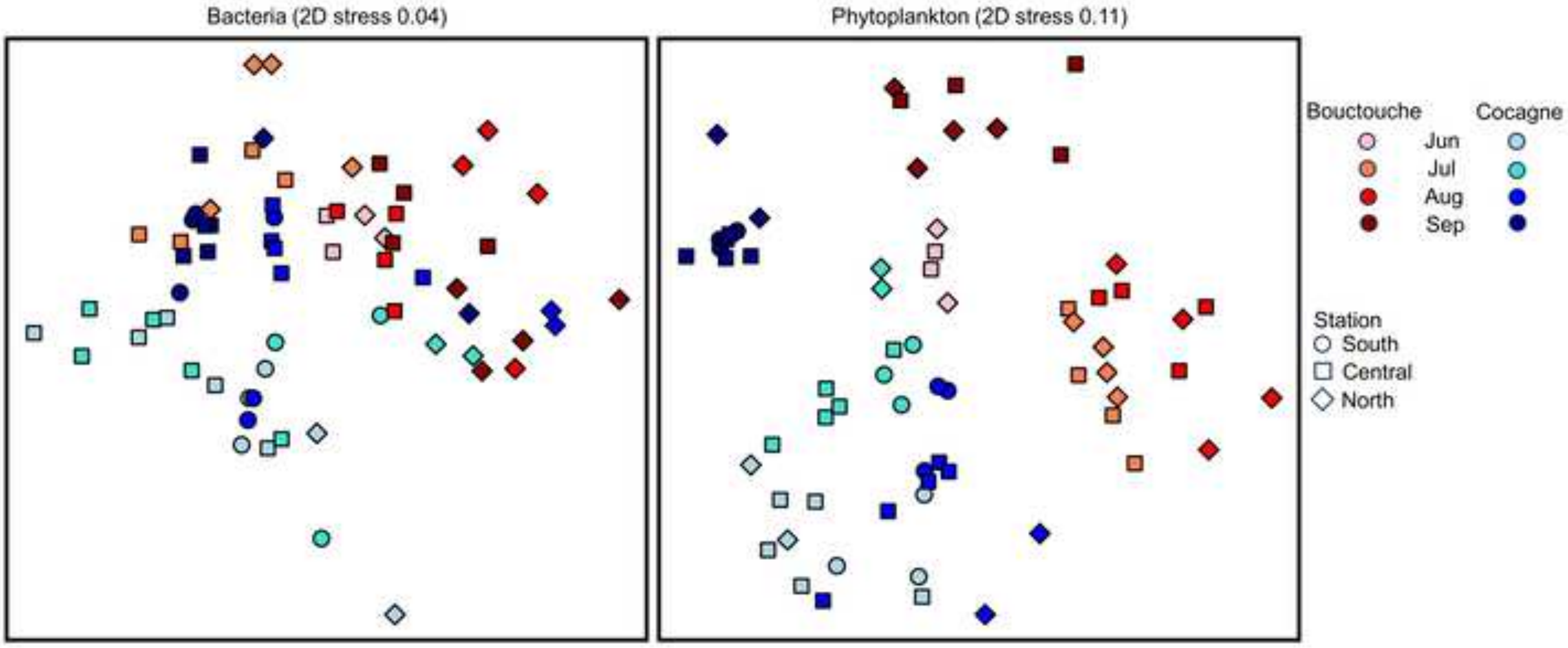

Fig10

Click here to access/download;Figure;Fig10.tif

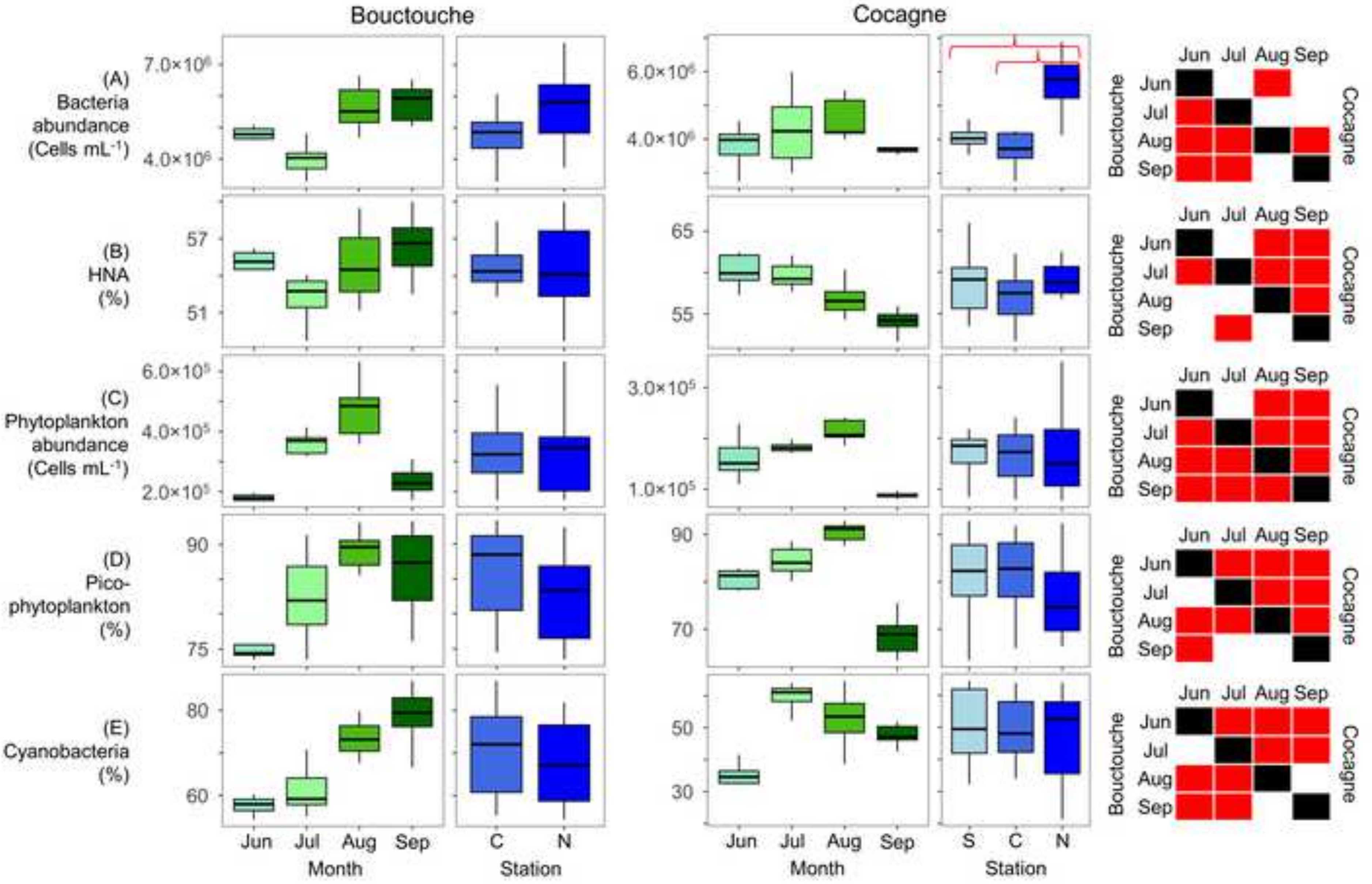

Fig11

Click here to access/download;Figure;Fig11.tif

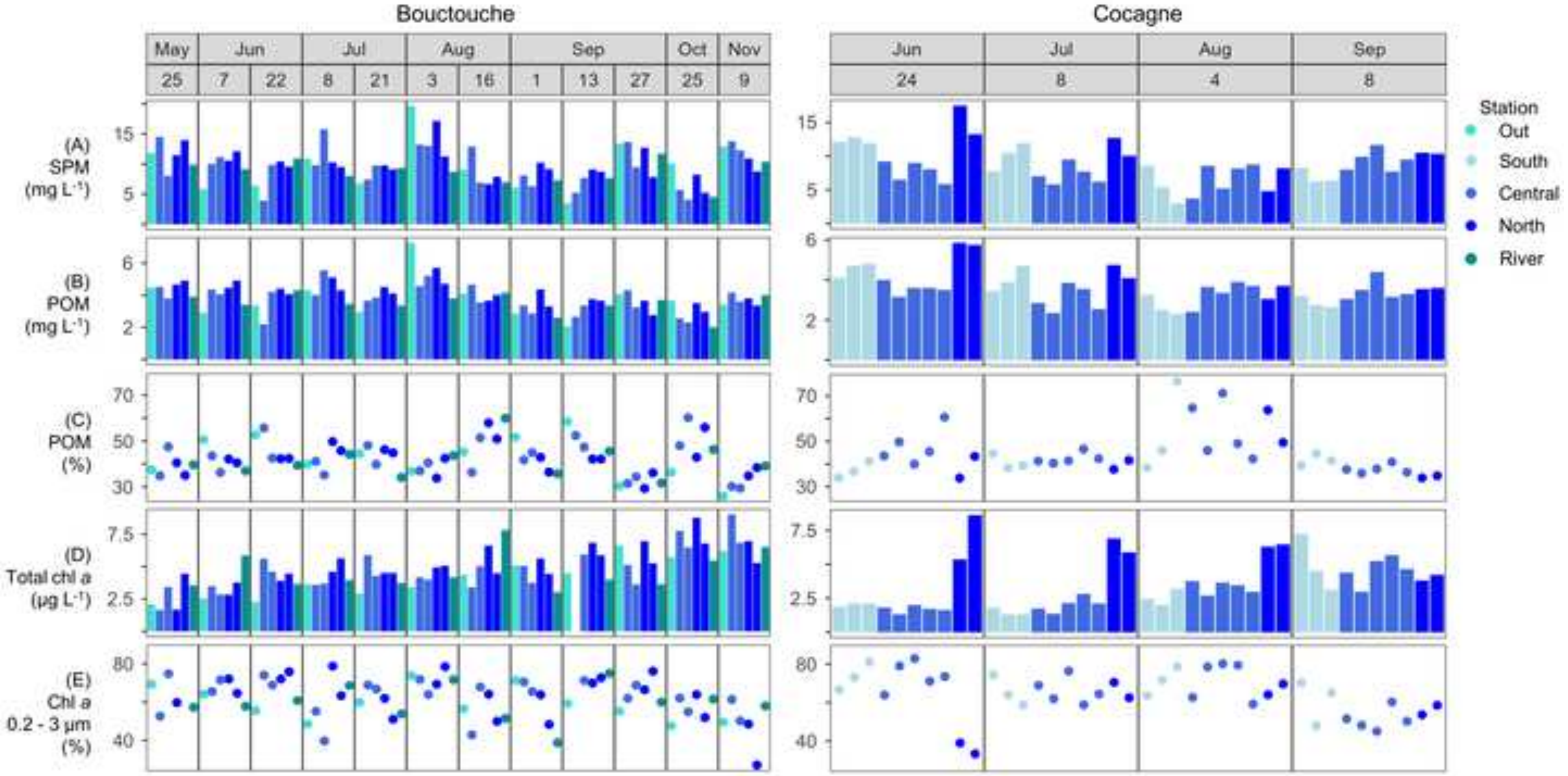

Fig12

Click here to access/download;Figure;Fig12.tif

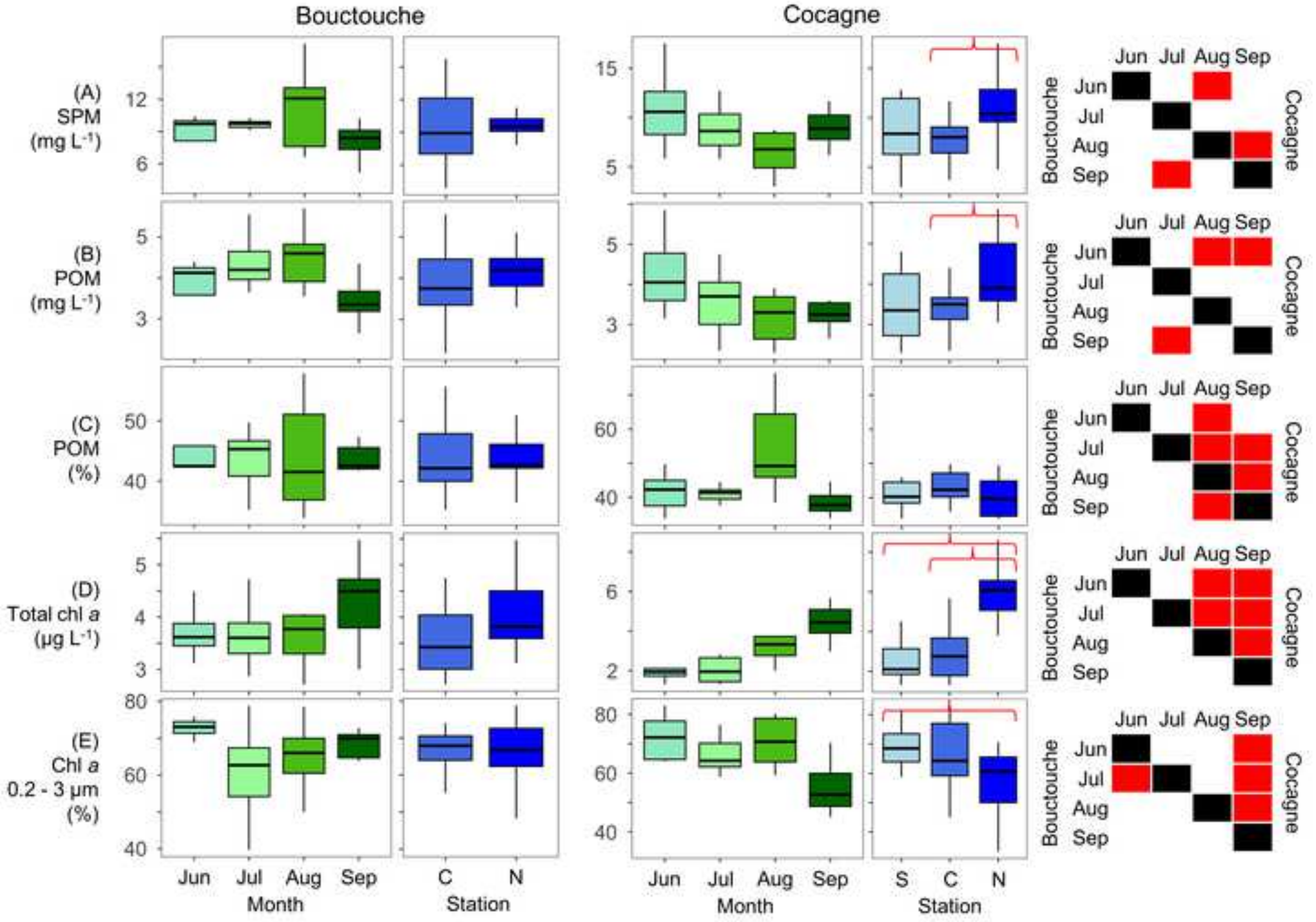

Fig13

Click here to access/download;Figure;Fig13.tif

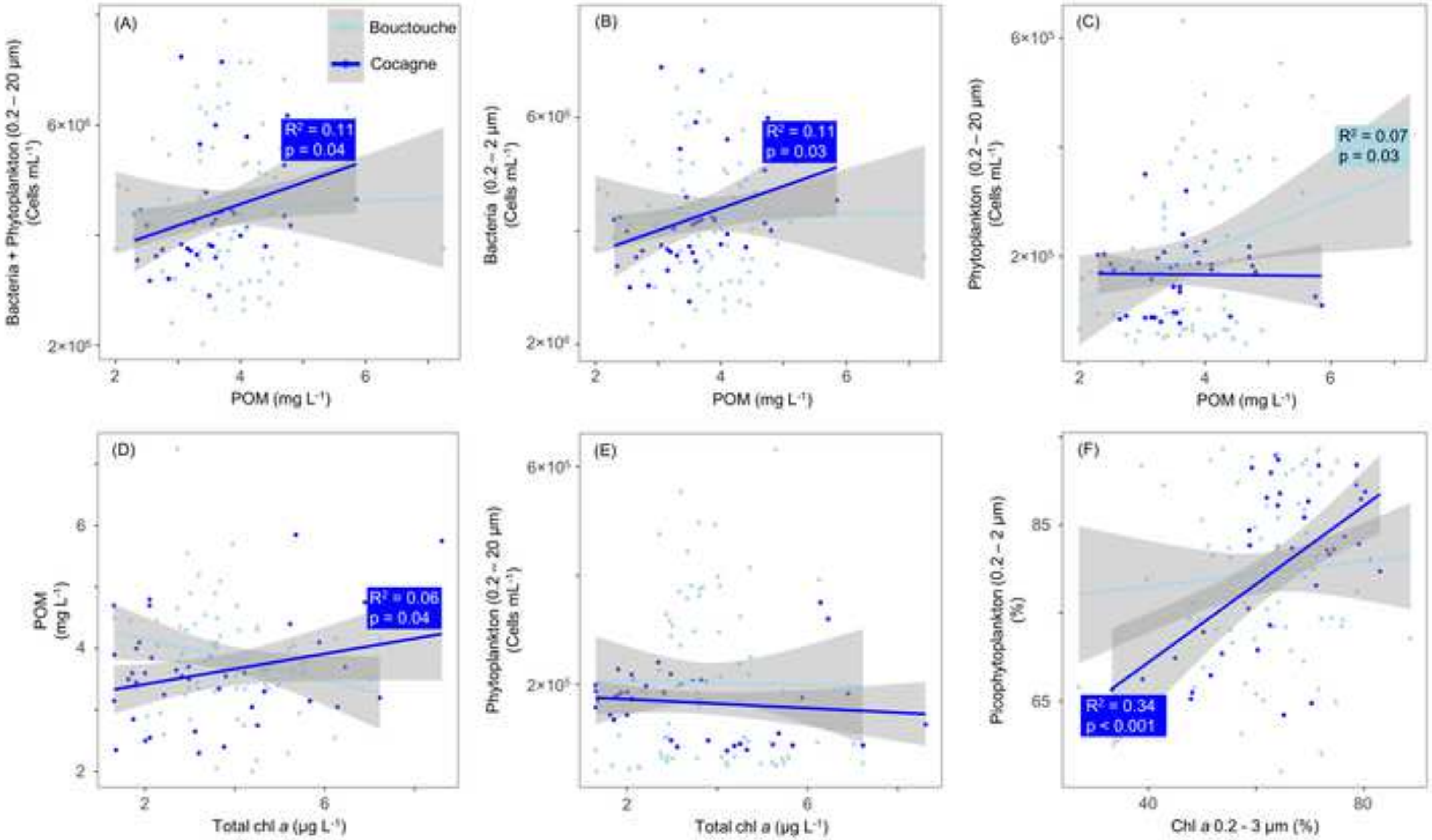

S1 Fig

Click here to access/download
Supporting Information
S1 Fig.tif
